# On *f*-Divergences: Integral Representations, Local Behavior, and Inequalities

**DOI:** 10.3390/e20050383

**Published:** 2018-05-19

**Authors:** Igal Sason

**Affiliations:** Department of Electrical Engineering, Technion-Israel Institute of Technology, Haifa 3200003, Israel; sason@ee.technion.ac.il; Tel.: +972-4-8294699

**Keywords:** DeGroot statistical information, *f*-divergences, local behavior, relative information spectrum, Rényi divergence

## Abstract

This paper is focused on *f*-divergences, consisting of three main contributions. The first one introduces integral representations of a general *f*-divergence by means of the relative information spectrum. The second part provides a new approach for the derivation of *f*-divergence inequalities, and it exemplifies their utility in the setup of Bayesian binary hypothesis testing. The last part of this paper further studies the local behavior of *f*-divergences.

## 1. Introduction

Probability theory, information theory, learning theory, statistical signal processing and other related disciplines, greatly benefit from non-negative measures of dissimilarity (a.k.a. divergence measures) between pairs of probability measures defined on the same measurable space (see, e.g., [1,2,3,4,5,6,7]). An axiomatic characterization of information measures, including divergence measures, was provided by Csiszár [8]. Many useful divergence measures belong to the set of *f*-divergences, independently introduced by Ali and Silvey [9], Csiszár [10,11,12,13], and Morimoto [14] in the early sixties. The family of *f*-divergences generalizes the relative entropy (a.k.a. the Kullback- Leibler divergence) while also satisfying the data processing inequality among other pleasing properties (see, e.g., [3] and references therein).

Integral representations of *f*-divergences serve to study properties of these information measures, and they are also used to establish relations among these divergences. An integral representation of *f*-divergences, expressed by means of the DeGroot statistical information, was provided in [3] with a simplified proof in [15]. The importance of this integral representation stems from the operational meaning of the DeGroot statistical information [16], which is strongly linked to Bayesian binary hypothesis testing. Some earlier specialized versions of this integral representation were introduced in [17,18,19,20,21], and a variation of it also appears in [22] Section 5.B. Implications of the integral representation of *f*-divergences, by means of the DeGroot statistical information, include an alternative proof of the data processing inequality, and a study of conditions for the sufficiency or ε-deficiency of observation channels [3,15].

Since many distance measures of interest fall under the paradigm of an *f*-divergence [23], bounds among *f*-divergences are very useful in many instances such as the analysis of rates of convergence and concentration of measure bounds, hypothesis testing, testing goodness of fit, minimax risk in estimation and modeling, strong data processing inequalities and contraction coefficients, etc. Earlier studies developed systematic approaches to obtain *f*-divergence inequalities while dealing with pairs of probability measures defined on arbitrary alphabets. A list of some notable existing *f*-divergence inequalities is provided, e.g., in [22] Section 1 and [23] Section 3. State-of-the-art techniques which serve to derive bounds among *f*-divergences include:

(1) Moment inequalities which rely on log-convexity arguments ([22] Section 5.D, [24,25,26,27,28]);
(2) Inequalities which rely on a characterization of the exact locus of the joint range of *f*-divergences [29];
(3) *f*-divergence inequalities via functional domination ([22] Section 3, [30,31,32]);
(4) Sharp *f*-divergence inequalities by using numerical tools for maximizing or minimizing an *f*-divergence subject to a finite number of constraints on other *f*-divergences [33];
(5) Inequalities which rely on powers of *f*-divergences defining a distance [34,35,36,37];
(6) Vajda and Pinsker-type inequalities for *f*-divergences ([4,10,13,22] Sections 6–7, [38,39]);
(7) Bounds among *f*-divergences when the relative information is bounded ([22] Sections 4–5, [40,41,42,43,44,45,46,47]), and reverse Pinsker inequalities ([22] Section 6, [40,48]);
(8) Inequalities which rely on the minimum of an *f*-divergence for a given total variation distance and related bounds [4,33,37,38,49,50,51,52,53];
(9) Bounds among *f*-divergences (or functions of *f*-divergences such as the Rényi divergence) via integral representations of these divergence measures [22] Section 8;
(10) Inequalities which rely on variational representations of *f*-divergences (e.g., [54] Section 2).

Following earlier studies of the local behavior of *f*-divergences and their asymptotic properties (see related results by Csiszár and Shields [55] Theorem 4.1, Pardo and Vajda [56] Section 3, and Sason and Vérdu [22] Section 3.F), it is known that the local behavior of *f*-divergences scales, such as the chi-square divergence (up to a scaling factor which depends on *f*) provided that the first distribution approaches the reference measure in a certain strong sense. The study of the local behavior of *f*-divergences is an important aspect of their properties, and we further study it in this work.

This paper considers properties of *f*-divergences, while first introducing in Section 2 the basic definitions and notation needed, and in particular the various measures of dissimilarity between probability measures used throughout this paper. The presentation of our new results is then structured as follows:

Section 3 is focused on the derivation of new integral representations of *f*-divergences, expressed as a function of the relative information spectrum of the pair of probability measures, and the convex function *f*. The novelty of Section 3 is in the unified approach which leads to integral representations of *f*-divergences by means of the relative information spectrum, where the latter cumulative distribution function plays an important role in information theory and statistical decision theory (see, e.g., [7,54]). Particular integral representations of the type of results introduced in Section 3 have been recently derived by Sason and Verdú in a case-by-case basis for some *f*-divergences (see [22] Theorems 13 and 32), while lacking the approach which is developed in Section 3 for general *f*-divergences. In essence, an *f*-divergence $D_{f}\left(P∥Q\right)$ is expressed in Section 3 as an inner product of a simple function of the relative information spectrum (depending only on the probability measures *P* and *Q*), and a non-negative weight function $\omega_{f}:\left(0,\infty \right)\mapsto \left[0,\infty \right)$ which only depends on *f*. This kind of representation, followed by a generalized result, serves to provide new integral representations of various useful *f*-divergences. This also enables in Section 3 to characterize the interplay between the DeGroot statistical information (or between another useful family of *f*-divergence, named the $E_{\gamma }$ divergence with γ≥1) and the relative information spectrum.

Section 4 provides a new approach for the derivation of *f*-divergence inequalities, where an arbitrary *f*-divergence is lower bounded by means of the $E_{\gamma }$ divergence [57] or the DeGroot statistical information [16]. The approach used in Section 4 yields several generalizations of the Bretagnole-Huber inequality [58], which provides a closed-form and simple upper bound on the total variation distance as a function of the relative entropy; the Bretagnole-Huber inequality has been proved to be useful, e.g., in the context of lower bounding the minimax risk in non-parametric estimation (see, e.g., [5] pp. 89–90, 94), and in the problem of density estimation (see, e.g., [6] Section 1.6). Although Vajda’s tight lower bound in [59] is slightly tighter everywhere than the Bretagnole-Huber inequality, our motivation for the generalization of the latter bound is justified later in this paper. The utility of the new inequalities is exemplified in the setup of Bayesian binary hypothesis testing.

Section 5 finally derives new results on the local behavior of *f*-divergences, i.e., the characterization of their scaling when the pair of probability measures are sufficiently close to each other. The starting point of our analysis in Section 5 relies on the analysis in [56] Section 3, regarding the asymptotic properties of *f*-divergences.

The reading of Section 3, Section 4 and Section 5 can be done in any order since the analysis in these sections is independent.

## 2. Preliminaries and Notation

We assume throughout that the probability measures *P* and *Q* are defined on a common measurable space (A,F), and P≪Q denotes that *P* is absolutely continuous with respect to *Q*, namely there is no event F∈F such that P(F)>0=Q(F).

**Definition** **1.** *The relative information provided by*a∈A*according to*(P,Q), *where*P≪Q, *is given by*

(1) $$\begin{array}{c} {ı}_{P∥Q}\left(a\right):=\log\;\frac{dP}{dQ}\;\left(a\right). \end{array}$$

*More generally, even if*P¬≪Q, *let R be an arbitrary dominating probability measure such that*P,Q≪R*(e.g.,*$R=\frac{1}{2}\left(P+Q\right)$*); irrespectively of the choice of R, the relative information is defined to be*

(2) $$\begin{array}{c} {ı}_{P∥Q}\left(a\right):={ı}_{P∥R}\left(a\right)-{ı}_{Q∥R}\left(a\right),\;a\in A. \end{array}$$

*The following asymmetry property follows from (2):*

(3) $$\begin{array}{c} {ı}_{P∥Q}=-{ı}_{Q∥P}. \end{array}$$

**Definition** **2.** *The relative information spectrum is the cumulative distribution function*

(4) $$\begin{array}{c} F_{P∥Q}\left(x\right)=P\left[{ı}_{P∥Q}\left(X\right)\leq x\right],\;x\in R,\;X∼P. \end{array}$$

*The relative entropy is the expected valued of the relative information when it is distributed according to P:*

(5) $$\begin{array}{c} D\left(P∥Q\right):=E\left[{ı}_{P∥Q}\left(X\right)\right],\;X∼P. \end{array}$$

Throughout this paper, C denotes the set of convex functions f:(0,∞)↦R with f(1)=0. Hence, the function f≡0 is in C; if f∈C, then af∈C for all a>0; and if f,g∈C, then f+g∈C. We next provide a general definition for the family of *f*-divergences (see [3] p. 4398).

**Definition** **3** (*f*-divergence [9,10,12])**.** *Let P and Q be probability measures, let μ be a dominating measure of P and Q (i.e.,* P,Q≪μ; *e.g.,* μ=P+Q*), and let* $p:=\frac{dP}{d\mu }$ *and* $q:=\frac{dQ}{d\mu }$. *The f-divergence from P to Q is given, independently of μ, by*

(6) $$\begin{array}{c} D_{f}\left(P∥Q\right):=\int q\;f\left(\frac{p}{q}\right)\;d\mu , \end{array}$$

*where*

(7) $$\begin{array}{c} f\left(0\right):=\lim_{t↓0}\;f\left(t\right), \end{array}$$

(8) $$\begin{array}{c} 0f\left(\frac{0}{0}\right):=0, \end{array}$$

(9) $$\begin{array}{c} 0f\left(\frac{a}{0}\right):=\lim_{t↓0}\;tf\left(\frac{a}{t}\right)=a\lim_{u\to \infty }\frac{f(u)}{u},\;a>0. \end{array}$$

We rely in this paper on the following properties of *f*-divergences:

**Proposition** **1.** *Let*f,g∈C. *The following conditions are equivalent:*

(1) (10) $$\begin{array}{c} D_{f}\left(P∥Q\right)=D_{g}\left(P∥Q\right),\;\forall \;P,Q; \end{array}$$
(2) *there exists a constant*c∈R*such that*
  (11) $$\begin{array}{c} f(t)-g(t)=c\;(t-1),\;\forall \;t\in (0,\infty ). \end{array}$$

**Proposition** **2.** *Let*f∈C, *and let*$f^{*}:\left(0,\infty \right)\mapsto R$*be the conjugate function, given by*

(12) $$\begin{array}{c} f^{*}\left(t\right)=t\;f\left(\frac{1}{t}\right) \end{array}$$

*for*t>0. *Then,*$f^{*}\in C$; $f^{**}=f$, *and for every pair of probability measures*(P,Q),

(13) $$\begin{array}{c} D_{f}\left(P∥Q\right)=D_{f^{*}}\left(Q∥P\right). \end{array}$$

By an analytic extension of $f^{*}$ in (12) at t=0, let

(14) $$\begin{array}{c} f^{*}\left(0\right):=\lim_{t↓0}f^{*}\left(t\right)=\lim_{u\to \infty }\frac{f(u)}{u}. \end{array}$$

Note that the convexity of $f^{*}$ implies that $f^{*}\left(0\right)\in \left(-\infty ,\infty \right]$. In continuation to Definition 3, we get

(15)Df(P∥Q)=∫qfpqdμ(16)=∫{pq>0}qfpqdμ+Q(p=0)f(0)+P(q=0)f∗(0)

with the convention in (16) that 0·∞=0, We refer in this paper to the following *f*-divergences:

(1) *Relative entropy*:
  (17) $$\begin{array}{cc} D(P∥Q) & =D_{f}\left(P∥Q\right). \end{array}$$
  with
  (18) $$\begin{array}{c} f(t)=t\;\log t,\;t>0. \end{array}$$
(2) *Jeffrey’s divergence [60]*:
  (19) $$\begin{array}{cc} J(P∥Q) & :=D(P∥Q)+D(Q∥P) \end{array}$$
  (20) $$\begin{array}{cc} & \;=D_{f}\left(P∥Q\right) \end{array}$$
  with
  (21) $$\begin{array}{c} f(t)=(t-1)\;\log t,\;t>0. \end{array}$$
(3) *Hellinger divergence of order*α∈(0,1)∪(1,∞) [2] Definition 2.10:
  (22) $$\begin{array}{c} H_{\alpha }\left(P∥Q\right)=D_{f_{\alpha }}\left(P∥Q\right) \end{array}$$
  with
  (23) $$\begin{array}{c} f_{\alpha }\left(t\right)=\frac{t^{\alpha }-1}{\alpha -1},\;t>0. \end{array}$$
  Some of the significance of the Hellinger divergence stems from the following facts:
  - The analytic extension of $H_{\alpha }\left(P∥Q\right)$ at α=1 yields
    (24) $$\begin{array}{c} D\left(P∥Q\right)=H_{1}\left(P∥Q\right)\;\log e. \end{array}$$
  - The *chi-squared divergence* [61] is the second order Hellinger divergence (see, e.g., [62] p. 48), i.e.,
    (25) $$\begin{array}{c} \chi^{2}\left(P∥Q\right)=H_{2}\left(P∥Q\right). \end{array}$$
    Note that, due to Proposition 1,
    (26) $$\begin{array}{c} \chi^{2}\left(P∥Q\right)=D_{f}\left(P∥Q\right), \end{array}$$
    where f:(0,∞)↦R can be defined as
    (27) $$\begin{array}{c} f\left(t\right)={\left(t-1\right)}^{2},\;t>0. \end{array}$$
  - The *squared Hellinger distance* (see, e.g., [62] p. 47), denoted by $H^{2}\left(P∥Q\right)$, satisfies the identity
    (28) $$\begin{array}{c} H^{2}\left(P∥Q\right)=\frac{1}{2}\;H_{\frac{1}{2}}\left(P∥Q\right). \end{array}$$
  - The *Bhattacharyya distance* [63], denoted by B(P∥Q), satisfies
    (29) $$\begin{array}{c} B\left(P∥Q\right)=\log\frac{1}{1-H^{2}\left(P∥Q\right)}. \end{array}$$
  - The *Rényi divergence* of order α∈(0,1)∪(1,∞) is a one-to-one transformation of the Hellinger divergence of the same order [11] (14):
    (30) $$\begin{array}{c} D_{\alpha }\left(P∥Q\right)=\frac{1}{\alpha -1}\;\log\left(1+\left(\alpha -1\right)\;H_{\alpha }\left(P∥Q\right)\right). \end{array}$$
  - The *Alpha-divergence* of order α, as it is defined in [64] and ([65] (4)), is a generalized relative entropy which (up to a scaling factor) is equal to the Hellinger divergence of the same order α. More explicitly,
    (31) $$\begin{array}{c} D_{A}^{\left(\alpha \right)}\left(P∥Q\right)=\frac{1}{\alpha }\;H_{\alpha }\left(P∥Q\right), \end{array}$$
    where $D_{A}^{\left(\alpha \right)}\left(\cdot ∥\cdot \right)$ denotes the Alpha-divergence of order α. Note, however, that the Beta and Gamma-divergences in [65], as well as the generalized divergences in [66,67], are not *f*-divergences in general.
(4) $\chi^{s}$*divergence* for s≥1 [2] (2.31), and the *total variation distance*: The function
  (32) $$\begin{array}{c} f_{s}\left(t\right)={\left|t-1\right|}^{s},\;t>0 \end{array}$$
  results in
  (33) $$\begin{array}{cc} \chi^{s}\left(P∥Q\right) & =D_{f_{s}}\left(P∥Q\right). \end{array}$$
  Specifically, for s=1, let
  (34) $$\begin{array}{c} f\left(t\right):=f_{1}\left(t\right)=\left|t-1\right|,\;t>0, \end{array}$$
  and the total variation distance is expressed as an *f*-divergence:
  (35) $$\begin{array}{cc} \left|P-Q\right| & =D_{f}\left(P∥Q\right). \end{array}$$
(5) *Triangular Discrimination [39] (a.k.a. Vincze-Le Cam distance)*:
  (36) $$\begin{array}{c} \Delta \left(P∥Q\right)=D_{f}\left(P∥Q\right) \end{array}$$
  with
  (37) $$\begin{array}{c} f\left(t\right)=\frac{{\left(t-1\right)}^{2}}{t+1},\;t>0. \end{array}$$
  Note that
  (38) $$\begin{array}{cc} \frac{1}{2}\;\Delta \left(P∥Q\right) & =\chi^{2}\left(P\;∥\;\frac{1}{2}P+\frac{1}{2}Q\right)=\chi^{2}\left(Q\;∥\;\frac{1}{2}P+\frac{1}{2}Q\right). \end{array}$$
(6) *Lin’s measure* [68] (4.1):
  (39)Lθ(P∥Q):=HθP+(1−θ)Q−θH(P)−(1−θ)H(Q)(40)=θDP∥θP+(1−θ)Q+(1−θ)DQ∥θP+(1−θ)Q,
  for θ∈[0,1]. This measure can be expressed by the following *f*-divergence:
  (41) $$\begin{array}{c} L_{\theta }\left(P∥Q\right)=D_{f_{\theta }}\left(P∥Q\right), \end{array}$$
  with
  (42) $$\begin{array}{c} f_{\theta }\left(t\right):=\theta t\log t-\left(\theta t+1-\theta \right)\;\log\left(\theta t+1-\theta \right),\;t>0. \end{array}$$
  The special case of (41) with $\theta =\frac{1}{2}$ gives the *Jensen-Shannon divergence* (a.k.a. capacitory discrimination):
  (43)JS(P∥Q):=L12(P∥Q)(44)=12DP∥12P+12Q+12DQ∥12P+12Q.
(7) $E_{\gamma }$*divergence* [57] p. 2314: For γ≥1,
  (45)Eγ(P∥Q):=maxU∈FP(U)−γQ(U)(46)=P[ıP∥Q(X)>logγ]−γP[ıP∥Q(Y)>logγ]
  with X∼P and Y∼Q, and where (46) follows from the Neyman-Pearson lemma. The $E_{\gamma }$ divergence can be identified as an *f*-divergence:
  (47) $$\begin{array}{c} E_{\gamma }\left(P∥Q\right)=D_{f_{\gamma }}\left(P∥Q\right) \end{array}$$
  with
  (48) $$\begin{array}{c} f_{\gamma }\left(t\right):={\left(t-\gamma \right)}^{+},\;t>0 \end{array}$$
  where ${\left(x\right)}^{+}:=\max\left\{x,0\right\}$. The following relation to the total variation distance holds:
  (49) $$\begin{array}{c} E_{1}\left(P∥Q\right)=\frac{1}{2}\;\left|P-Q\right|. \end{array}$$
(8) *DeGroot statistical information* [3,16]: For ω∈(0,1),
  (50) $$\begin{array}{c} I_{\omega }\left(P∥Q\right)=D_{\phi_{\omega }}\left(P∥Q\right) \end{array}$$
  with
  (51) $$\begin{array}{c} \phi_{\omega }\left(t\right)=\min\left\{\omega ,1-\omega \right\}-\min\left\{\omega t,1-\omega \right\},\;t>0. \end{array}$$
  The following relation to the total variation distance holds:
  (52) $$\begin{array}{c} I_{\frac{1}{2}}\left(P∥Q\right)=\frac{1}{4}\;\left|P-Q\right|, \end{array}$$
  and the DeGroot statistical information and the $E_{\gamma }$ divergence are related as follows [22] (384):
  (53) $$\begin{array}{c} \begin{array}{c} I_{\omega }\left(P∥Q\right)=\{\begin{array}{cc} \omega \;E_{\frac{1-\omega }{\omega }}\left(P∥Q\right), & \;\omega \in \left(0,\frac{1}{2}\right],\\ \left(1-\omega \right)\;E_{\frac{\omega }{1-\omega }}\left(Q∥P\right), & \;\omega \in (\frac{1}{2},1). \end{array} \end{array} \end{array}$$

## 3. New Integral Representations of f-Divergences

The main result in this section provides new integral representations of *f*-divergences as a function of the relative information spectrum (see Definition 2). The reader is referred to other integral representations (see [15] Section 2, [4] Section 5, [22] Section 5.B, and references therein), expressing a general *f*-divergence by means of the DeGroot statistical information or the $E_{\gamma }$ divergence.

**Lemma** **1.** *Let*f∈C*be a strictly convex function at 1. Let*g:R↦R*be defined as*

(54) $$\begin{array}{c} g\left(x\right):=\exp\left(-x\right)\;f\left(\exp(x)\right)-f_{+}^{\prime }\left(1\right)\;\left(1-\exp(-x)\right),\;x\in R \end{array}$$

*where*$f_{+}^{\prime }\left(1\right)$*denotes the right-hand derivative of f at 1 (due to the convexity of f on*(0,∞), *it exists and it is finite). Then, the function g is non-negative, it is strictly monotonically decreasing on*(−∞,0], *and it is strictly monotonically increasing on*[0,∞)*with*g(0)=0.

**Proof.**  For any function u∈C, let $\tilde{u}\in C$ be given by

(55) $$\begin{array}{c} \tilde{u}\left(t\right)=u\left(t\right)-u_{+}^{\prime }\left(1\right)\left(t-1\right),\;t\in \left(0,\infty \right), \end{array}$$

and let $u^{*}\in C$ be the conjugate function, as given in (12). The function *g* in (54) can be expressed in the form

(56) $$\begin{array}{c} g\left(x\right)={\left(\tilde{f}\right)}^{*}\left(\exp(-x)\right),\;x\in R, \end{array}$$

as it is next verified. For t>0, we get from (12) and (55),

(57) $$\begin{array}{c} {\left(\tilde{f}\right)}^{*}\left(t\right)=t\tilde{f}\left(\frac{1}{t}\right)=tf\left(\frac{1}{t}\right)+f_{+}^{\prime }\left(1\right)\;\left(t-1\right), \end{array}$$

and the substitution t:=exp(−x) for x∈R yields (56) in view of (54).

By assumption, f∈C is strictly convex at 1, and therefore these properties are inherited to $\tilde{f}$. Since also $\tilde{f}\left(1\right)={\tilde{f}}^{\prime }\left(1\right)=0$, it follows from [3] Theorem 3 that both $\tilde{f}$ and ${\tilde{f}}^{*}$ are non-negative on (0,∞), and they are also strictly monotonically decreasing on (0,1]. Hence, from (12), it follows that the function ${\left(\tilde{f}\right)}^{*}$ is strictly monotonically increasing on [1,∞). Finally, the claimed properties of the function *g* follow from (56), and in view of the fact that the function ${\left(\tilde{f}\right)}^{*}$ is non-negative with ${\left(\tilde{f}\right)}^{*}\left(1\right)=0$, strictly monotonically decreasing on (0,1] and strictly monotonically increasing on [1,∞). ☐

**Lemma** **2.** *Let*f∈C*be a strictly convex function at 1, and let*g:R↦R*be as in* (54)*. Let*

(58) $$\begin{array}{cc} a & :=\lim_{x\to \infty }g\left(x\right)\in \left(0,\infty \right], \end{array}$$

(59) $$\begin{array}{cc} b & :=\lim_{x\to -\infty }g\left(x\right)\in \left(0,\infty \right], \end{array}$$

*and let* $\ell_{1}:\left[0,a\right)\mapsto \left[0,\infty \right)$ *and* $\ell_{2}:\left[0,b\right)\mapsto \left(-\infty ,0\right]$ *be the two inverse functions of g. Then,*

(60) $$\begin{array}{c} D_{f}\left(P∥Q\right)=\int_{0}^{a}\left[1-F_{P∥Q}\left(\ell_{1}\left(t\right)\right)\right]\;dt+\int_{0}^{b}F_{P∥Q}\left(\ell_{2}\left(t\right)\right)\;dt. \end{array}$$

**Proof.**  In view of Lemma 1, it follows that $\ell_{1}:\left[0,a\right)\mapsto \left[0,\infty \right)$ is strictly monotonically increasing and $\ell_{2}:\left[0,b\right)\mapsto \left(-\infty ,0\right]$ is strictly monotonically decreasing with $\ell_{1}\left(0\right)=\ell_{2}\left(0\right)=0$.

Let X∼P, and let $V:=\exp\left({ı}_{P∥Q}\left(X\right)\right)$. Then, we have

(61)Df(P∥Q)=Df˜(P∥Q)(62)=D(f˜)∗(Q∥P)(63)=∫(f˜)∗expıQ∥P(x)dP(x)(64)=∫(f˜)∗exp−ıP∥Q(x)dP(x)(65)=∫gıP∥Q(x)dP(x)(66)=Eg(V)(67)=∫0∞Pg(V)>tdt(68)=∫0aPV≥0,g(V)>tdt+∫0bPV<0,g(V)>tdt(69)=∫0aPV>ℓ1(t)dt+∫0bPV≤ℓ2(t)dt(70)=∫0a1−FP∥Qℓ1(t)dt+∫0bFP∥Qℓ2(t)dt

where (61) relies on Proposition 1; (62) relies on Proposition 2; (64) follows from (3); (65) follows from (56); (66) holds by the definition of the random variable *V*; (67) holds since, in view of Lemma 1, Z:=g(V)≥0, and $E\left[Z\right]=\int_{0}^{\infty }P\left[Z>t\right]\;dt$ for any non-negative random variable *Z*; (68) holds in view of the monotonicity properties of *g* in Lemma 1, the definition of *a* and *b* in (58) and (59), and by expressing the event {g(V)>t} as a union of two disjoint events; (69) holds again by the monotonicity properties of *g* in Lemma 1, and by the definition of its two inverse functions $\ell_{1}$ and $\ell_{2}$ as above; in (67)–(69) we are free to substitute > by ≥, and < by ≤; finally, (70) holds by the definition of the relative information spectrum in (4). ☐

**Remark** **1.** *The function*g:R↦R*in* (54) *is invariant to the mapping* f(t)↦f(t)+c(t−1), *for* t>0, *with an arbitrary* c∈R. *This invariance of g (and, hence, also the invariance of its inverse functions* $\ell_{1}$ *and* $\ell_{2}$) *is well expected in view of Proposition 1 and Lemma 2*.

**Example** **1.** *For the chi-squared divergence in* (26)*, letting f be as in* (27)*, it follows from* (54) *that*

(71) $$\begin{array}{c} g\left(x\right)=4\sinh^{2}\left(\frac{1}{2\log e}\;x\right),\;x\in R, \end{array}$$

*which yields, from* (58) *and (59),* a=b=∞. *Calculation of the two inverse functions of g, as defined in Lemma 2, yields the following closed-form expression*:

(72) $$\begin{array}{cc} \ell_{1,2}\left(u\right) & =\pm 2\log\left(\frac{u+\sqrt{u+4}}{2}\right),\;u\geq 0. \end{array}$$

*Substituting* (72) *into* (60) *provides an integral representation of* $\chi^{2}\left(P∥Q\right)$.

**Lemma** **3.** (73) $$\begin{array}{c} \int_{0}^{\infty }\frac{F_{P∥Q}\left(\log\beta \right)}{\beta^{2}}\;d\beta =1. \end{array}$$

**Proof.**  Let X∼P. Then, we have

(74)∫0∞FP∥Q(logβ)β2dβ=∫0∞1β2P[ıP∥Q(X)≤logβ]dβ(75)=∫0∞1β2PexpıQ∥P(X)≥1βdβ(76)=∫0∞PexpıQ∥P(X)≥udu(77)=EexpıQ∥P(X)(78)=1,

where (74) holds by (4); (75) follows from (3); (76) holds by the substitution $u:=\frac{1}{\beta }$; (77) holds since $\exp\left({ı}_{Q∥P}\left(X\right)\right)\geq 0$, and finally (78) holds since X∼P. ☐

**Remark** **2.** *Unlike Example 1, in general, the inverse functions*$\ell_{1}$*and*$\ell_{2}$*in Lemma 2 are not expressible in closed form, motivating our next integral representation in Theorem 1*.

The following theorem provides our main result in this section.

**Theorem** **1.** *The following integral representations of an f-divergence, by means of the relative information spectrum, hold:*

*(1)*  *Let*
  - f∈C*be differentiable on*(0,∞);
  - $w_{f}:\left(0,\infty \right)\mapsto \left[0,\infty \right)$*be the non-negative weight function given, for*β>0, *by*
    (79) $$\begin{array}{cc} w_{f}\left(\beta \right) & :=\frac{1}{\beta }\left|f^{\prime }\left(\beta \right)-\frac{f\left(\beta \right)+f^{\prime }\left(1\right)}{\beta }\right|; \end{array}$$
  - *the function* $G_{P∥Q}:\left(0,\infty \right)\mapsto \left[0,1\right]$ *be given by*
    (80) $$G_{P∥Q}\left(\beta \right):=\{\begin{array}{cc} 1-F_{P∥Q}\left(\log\beta \right), & \beta \in [1,\infty ),\\ F_{P∥Q}\left(\log\beta \right), & \beta \in (0,1). \end{array}$$
*Then,*
  (81) $$\begin{array}{c} D_{f}\left(P∥Q\right)=\langle w_{f},\;G_{P∥Q}\rangle =\int_{0}^{\infty }w_{f}\left(\beta \right)\;G_{P∥Q}\left(\beta \right)\;d\beta . \end{array}$$
*(2)*  *More generally, for an arbitrary*c∈R, *let*${\tilde{w}}_{f,c}:\left(0,\infty \right)\mapsto R$*be a modified real-valued function defined as*
  (82) $$\begin{array}{c} {\tilde{w}}_{f,c}\left(\beta \right):=w_{f}\left(\beta \right)+\frac{c}{\beta^{2}}\;\left(1\{\beta \geq 1\}-1\{0<\beta <1\}\right). \end{array}$$
*Then,*
  (83) $$\begin{array}{c} D_{f}\left(P∥Q\right)=\langle {\tilde{w}}_{f,c},\;G_{P∥Q}\rangle . \end{array}$$

**Proof.**  We start by proving the special integral representation in (81), and then extend our proof to the general representation in (83).

(1) We first assume an additional requirement that *f* is strictly convex at 1. In view of Lemma 2,
  (84) $$\begin{array}{cc} & \ell_{1}\left(g(u)\right)=u,\;u\in \left[0,\infty \right), \end{array}$$
  (85) $$\begin{array}{cc} & \ell_{2}\left(g(u)\right)=u,\;u\in \left(-\infty ,0\right]. \end{array}$$
  Since by assumption f∈C is differentiable on (0,∞) and strictly convex at 1, the function *g* in (54) is differentiable on R. In view of (84) and (85), substituting $t:=g\left(\log\beta \right)$ in (60) for β>0 implies that
  (86) $$\begin{array}{c} D_{f}\left(P∥Q\right)=\int_{1}^{\infty }\left[1-F_{P∥Q}\left(\log\beta \right)\right]\;{\bar{w}}_{f}\left(\beta \right)\;d\beta -\int_{0}^{1}F_{P∥Q}\left(\log\beta \right)\;{\bar{w}}_{f}\left(\beta \right)\;d\beta , \end{array}$$
  where ${\bar{w}}_{f}:\left(0,\infty \right)\mapsto R$ is given by
  (87)w¯f(β):=g′logββloge(88)=1βf′(β)−f(β)+f′(1)β
  for β>0, where (88) follows from (54). Due to the monotonicity properties of *g* in Lemma 1, (87) implies that ${\bar{w}}_{f}\left(\beta \right)\geq 0$ for β≥1, and ${\bar{w}}_{f}\left(\beta \right)<0$ for β∈(0,1). Hence, the weight function $w_{f}$ in (79) satisfies
  (89) $$\begin{array}{c} w_{f}\left(\beta \right)=\left|{\bar{w}}_{f}\left(\beta \right)\right|={\bar{w}}_{f}\left(\beta \right)\;\left(1\{\beta \geq 1\}-1\{0<\beta <1\}\right),\;\beta >0. \end{array}$$
  The combination of (80), (86) and (89) gives the required result in (81).
  We now extend the result in (81) when f∈C is differentiable on (0,∞), but not necessarily strictly convex at 1. To that end, let s:(0,∞)↦R be defined as
  (90) $$\begin{array}{c} s\left(t\right):=f\left(t\right)+\left(t^{2}-1\right),\;t>0. \end{array}$$
  This implies that s∈C is differentiable on (0,∞), and it is also strictly convex at 1. In view of the proof of (81) when *f* is strict convexity of *f* at 1, the application of this result to the function *s* in (90) yields
  (91) $$\begin{array}{c} D_{s}\left(P∥Q\right)=\langle w_{s},\;G_{P∥Q}\rangle . \end{array}$$
  In view of (6), (22), (23), (25) and (90),
  (92) $$\begin{array}{c} D_{s}\left(P∥Q\right)=D_{f}\left(P∥Q\right)+\chi^{2}\left(P∥Q\right); \end{array}$$
  from (79), (89), (90) and the convexity and differentiability of f∈C, it follows that the weight function $w_{s}\in \left(0,\infty \right)\mapsto \left[0,\infty \right)$ satisfies
  (93) $$\begin{array}{c} w_{s}\left(\beta \right)=w_{f}\left(\beta \right)+\left(1-\frac{1}{\beta^{2}}\right)\left(1\{\beta \geq 1\}-1\{0<\beta <1\}\right) \end{array}$$
  for β>0. Furthermore, by applying the result in (81) to the chi-squared divergence $\chi^{2}\left(P∥Q\right)$ in (25) whose corresponding function $f_{2}\left(t\right):=t^{2}-1$ for t>0 is strictly convex at 1, we obtain
  (94) $$\begin{array}{c} \chi^{2}\left(P∥Q\right)=\int_{0}^{\infty }\left(1-\frac{1}{\beta^{2}}\right)\left(1\{\beta \geq 1\}-1\{0<\beta <1\}\right)\;G_{P∥Q}\left(\beta \right)\;d\beta . \end{array}$$
  Finally, the combination of (91)–(94), yields $D_{f}\left(P∥Q\right)=\langle w_{f},\;G_{P∥Q}\rangle$; this asserts that (81) also holds by relaxing the condition that *f* is strictly convex at 1.
(2) In view of (80)–(82), in order to prove (83) for an arbitrary c∈R, it is required to prove the identity
  (95) $$\begin{array}{c} \int_{1}^{\infty }\frac{1-F_{P∥Q}\left(\log\beta \right)}{\beta^{2}}\;d\beta =\int_{0}^{1}\frac{F_{P∥Q}\left(\log\beta \right)}{\beta^{2}}\;d\beta . \end{array}$$
  Equality (95) can be verified by Lemma 3: by rearranging terms in (95), we get the identity in (73) (since $\int_{1}^{\infty }\frac{d\beta }{\beta^{2}}=1$). ☐

**Remark** **3.** *Due to the convexity of f, the absolute value in the right side of* (79) *is only needed for* β∈(0,1) *(see* (88) *and* (89)*). Also,* $w_{f}\left(1\right)=0$ *since* f(1)=0.

**Remark** **4.** *The weight function*$w_{f}$*only depends on f, and the function*$G_{P∥Q}$*only depends on the pair of probability measures P and Q. In view of Proposition 1, it follows that, for*f,g∈C, *the equality*$w_{f}=w_{g}$*holds on*(0,∞)*if and only if* (11) *is satisfied with an arbitrary constant* c∈R. *It is indeed easy to verify that* (11) *yields* $w_{f}=w_{g}$ *on* (0,∞).

**Remark** **5.** *An equivalent way to write*$G_{P∥Q}$*in* (80) *is*

(96) $$G_{P∥Q}\left(\beta \right)=\{\begin{array}{cc} P\left[\frac{dP}{dQ}\;\left(X\right)>\beta \right], & \beta \in [1,\infty )\\ P\left[\frac{dP}{dQ}\;\left(X\right)\leq \beta \right], & \beta \in (0,1) \end{array}$$

*where* X∼P. *Hence, the function* $G_{P∥Q}:\left(0,\infty \right)\mapsto \left[0,1\right]$ *is monotonically increasing in* (0,1), *and it is monotonically decreasing in* [1,∞); *note that this function is in general discontinuous at 1 unless* $F_{P∥Q}\left(0\right)=\frac{1}{2}$. *If* P≪≫Q, *then*

(97) $$\begin{array}{c} \lim_{\beta ↓0}G_{P∥Q}\left(\beta \right)=\lim_{\beta \to \infty }G_{P∥Q}\left(\beta \right)=0. \end{array}$$

*Note that if*P=Q, *then*$G_{P∥Q}$*is zero everywhere, which is consistent with the fact that*$D_{f}\left(P∥Q\right)=0$.

**Remark** **6.** *In the proof of Theorem 1-(1), the relaxation of the condition of strict convexity at 1 for a differentiable function f∈C is crucial, e.g., for the $\chi^{s}$ divergence with s>2. To clarify this claim, note that in view of* (32)*, the function $f_{s}:\left(0,\infty \right)\mapsto R$ is differentiable if s>1, and $f_{s}\in C$ with $f_{s}^{\prime }\left(1\right)=0$; however, $f_{s}^{\prime \prime }\left(1\right)=0$ if s>2, so $f_{s}$ in not strictly convex at 1 unless s∈[1,2].*

**Remark** **7.** *Theorem 1-(1) with c≠0 enables, in some cases, to simplify integral representations of f-divergences. This is next exemplified in the proof of Theorem 2.*

Theorem 1 yields integral representations for various *f*-divergences and related measures; some of these representations were previously derived by Sason and Verdú in [22] in a case by case basis, without the unified approach of Theorem 1. We next provide such integral representations. Note that, for some *f*-divergences, the function f∈C is not differentiable on (0,∞); hence, Theorem 1 is not necessarily directly applicable.

**Theorem** **2.** *The following integral representations hold as a function of the relative information spectrum:*

*(1)*  *Relative entropy [22] (219):*
  (98) $$\begin{array}{cc} \frac{1}{\log e}\;D\left(P∥Q\right) & =\int_{1}^{\infty }\frac{1-F_{P∥Q}\left(\log\beta \right)}{\beta }\;d\beta -\int_{0}^{1}\frac{F_{P∥Q}\left(\log\beta \right)}{\beta }\;d\beta . \end{array}$$
*(2)*  *Hellinger divergence of order α∈(0,1)∪(1,∞) [22] (434) and (437):*
  (99) $$H_{\alpha }\left(P∥Q\right)=\{\begin{array}{cc} \frac{1}{1-\alpha }-\int_{0}^{\infty }\beta^{\alpha -2}\;F_{P∥Q}\left(\log\beta \right)\;d\beta , & \;\alpha \in (0,1)\\ \int_{0}^{\infty }\beta^{\alpha -2}\left(1-F_{P∥Q}\left(\log\beta \right)\right)\;d\beta -\frac{1}{\alpha -1}, & \;\alpha \in (1,\infty ). \end{array}$$
  *In particular, the chi-squared divergence, squared Hellinger distance and Bhattacharyya distance satisfy*
  (100) $$\begin{array}{cc} \chi^{2}\left(P∥Q\right) & =\int_{0}^{\infty }\left(1-F_{P∥Q}\left(\log\beta \right)\right)\;d\beta -1; \end{array}$$
  (101) $$\begin{array}{cc} H^{2}\left(P∥Q\right) & =1-\frac{1}{2}\int_{0}^{\infty }\beta^{-\frac{3}{2}}\;F_{P∥Q}\left(\log\beta \right)\;d\beta ; \end{array}$$
  (102) $$\begin{array}{cc} B(P∥Q) & =\log2-\log\left(\int_{0}^{\infty }\beta^{-\frac{3}{2}}\;F_{P∥Q}\left(\log\beta \right)\;d\beta \right), \end{array}$$
  *where* (100) *appears in [22] (439).*
*(3)*  *Rényi divergence [22] (426) and (427): For α∈(0,1)∪(1,∞),*
  (103) $$D_{\alpha }\left(P∥Q\right)=\{\begin{array}{cc} \frac{1}{\alpha -1}\;\log\left(\left(1-\alpha \right)\int_{0}^{\infty }\beta^{\alpha -2}\;F_{P∥Q}\left(\log\beta \right)\;d\beta \right), & \;\alpha \in (0,1)\\ \frac{1}{\alpha -1}\;\log\left(\left(\alpha -1\right)\int_{0}^{\infty }\beta^{\alpha -2}\left(1-F_{P∥Q}\left(\log\beta \right)\right)\;d\beta \right), & \;\alpha \in (1,\infty ). \end{array}$$
*(4)*  *$\chi^{s}$ divergence: For s≥1*
  (104) $$\begin{array}{cc} \chi^{s}\left(P∥Q\right) & =\int_{1}^{\infty }\frac{1}{\beta }\left(s-1+\frac{1}{\beta }\right){\left(\beta -1\right)}^{s-1}\left(1-F_{P∥Q}\left(\log\beta \right)\right)\;d\beta \\ & +\int_{0}^{1}\frac{1}{\beta }\left(s-1+\frac{1}{\beta }\right){\left(1-\beta \right)}^{s-1}\;F_{P∥Q}\left(\log\beta \right)\;d\beta . \end{array}$$
  *In particular, the following identities hold for the total variation distance:*
  (105)|P−Q|=2∫1∞1−FP∥Q(logβ)β2dβ(106)=2∫01FP∥Q(logβ)β2dβ,
  *where* (105) *appears in [22] (214).*
*(5)*  *DeGroot statistical information:*
  (107) $$I_{w}\left(P∥Q\right)=\{\begin{array}{cc} \left(1-w\right)\int_{0}^{\frac{1-w}{w}}\frac{F_{P∥Q}\left(\log\beta \right)}{\beta^{2}}\;d\beta , & \;w\in (\frac{1}{2},1)\\ \left(1-w\right)\int_{\frac{1-w}{w}}^{\infty }\frac{1-F_{P∥Q}\left(\log\beta \right)}{\beta^{2}}\;d\beta , & \;w\in \left(0,\frac{1}{2}\right]. \end{array}$$
*(6)*  *Triangular discrimination:*
  (108) $$\begin{array}{cc} \Delta (P∥Q) & =4\int_{0}^{\infty }\frac{1-F_{P∥Q}\left(\log\beta \right)}{{\left(\beta +1\right)}^{2}}\;d\beta -2. \end{array}$$
*(7)*  *Lin’s measure: For θ∈[0,1],*
  (109) $$\begin{array}{cc} L_{\theta }\left(P∥Q\right) & =h\left(\theta \right)-\left(1-\theta \right)\int_{0}^{\infty }\frac{\log\left(1+\frac{\theta \beta }{1-\theta }\right)}{\beta^{2}}\;F_{P∥Q}\left(\log\beta \right)\;d\beta , \end{array}$$
  *where h:[0,1]↦[0,log2] denotes the binary entropy function. Specifically, the Jensen-Shannon divergence admits the integral representation:*
  (110) $$\begin{array}{cc} \mathrm{JS}(P∥Q) & =\log2-\int_{0}^{\infty }\frac{\log(\beta +1)}{2\beta^{2}}\;F_{P∥Q}\left(\log\beta \right)\;d\beta . \end{array}$$
*(8)*  *Jeffrey’s divergence:*
  (111) $$\begin{array}{cc} J(P∥Q) & =\int_{1}^{\infty }\left(1-F_{P∥Q}\left(\log\beta \right)\right)\left(\frac{\log e}{\beta }+\frac{\log\beta }{\beta^{2}}\right)\;d\beta \\ & \;-\int_{0}^{1}F_{P∥Q}\left(\log\beta \right)\;\left(\frac{\log e}{\beta }+\frac{\log\beta }{\beta^{2}}\right)\;d\beta . \end{array}$$
*(9)*  *$E_{\gamma }$ divergence: For γ≥1,*
  (112) $$\begin{array}{c} E_{\gamma }\left(P∥Q\right)=\gamma \int_{\gamma }^{\infty }\frac{1-F_{P∥Q}\left(\log\beta \right)}{\beta^{2}}\;d\beta . \end{array}$$

**Proof.**  See Appendix A. ☐

An application of (112) yields the following interplay between the $E_{\gamma }$ divergence and the relative information spectrum.

**Theorem** **3.** *Let X∼P, and let the random variable ${ı}_{P∥Q}\left(X\right)$ have no probability masses. Denote*

(113) $$\begin{array}{cc} & A_{1}:=\left\{E_{\gamma }\left(P∥Q\right):\gamma \geq 1\right\}, \end{array}$$

(114) $$\begin{array}{cc} & A_{2}:=\left\{E_{\gamma }\left(Q∥P\right):\gamma >1\right\}. \end{array}$$

*Then,*

- *$E_{\gamma }\left(P∥Q\right)$ is a continuously differentiable function of γ on (1,∞), and $E_{\gamma }^{\prime }\left(P∥Q\right)\leq 0$;*
- *the sets $A_{1}$ and $A_{2}$ determine, respectively, the relative information spectrum $F_{P∥Q}\left(\cdot \right)$ on [0,∞) and (−∞,0);*
- *for γ>1,*
  (115) $$\begin{array}{cc} & F_{P∥Q}\left(+\log\gamma \right)=1-E_{\gamma }\left(P∥Q\right)+\gamma E_{\gamma }^{\prime }\left(P∥Q\right), \end{array}$$
  (116) $$\begin{array}{cc} & F_{P∥Q}\left(-\log\gamma \right)=-E_{\gamma }^{\prime }\left(Q∥P\right), \end{array}$$
  (117)FP∥Q(0)=1−E1(P∥Q)+limγ↓1Eγ′(P∥Q)(118)=−limγ↓1Eγ′(Q∥P).

**Proof.**  We start by proving the first item. By our assumption, $F_{P∥Q}\left(\cdot \right)$ is continuous on R. Hence, it follows from (112) that $E_{\gamma }\left(P∥Q\right)$ is continuously differentiable in γ∈(1,∞); furthermore, (45) implies that $E_{\gamma }\left(P∥Q\right)$ is monotonically decreasing in γ, which yields $E_{\gamma }^{\prime }\left(P∥Q\right)\leq 0$.

We next prove the second and third items together. Let X∼P and Y∼Q. From (112), for γ>1,

(119) $$\begin{array}{c} \frac{d}{d\gamma }\left(\frac{E_{\gamma }\left(P∥Q\right)}{\gamma }\right)=-\frac{1-F_{P∥Q}\left(\log\gamma \right)}{\gamma^{2}}, \end{array}$$

which yields (115). Due to the continuity of $F_{P∥Q}\left(\cdot \right)$, it follows that the set $A_{1}$ determines the relative information spectrum on [0,∞).

To prove (116), we have

(120)Eγ(Q∥P)=P[ıQ∥P(Y)>logγ]−γP[ıQ∥P(X)>logγ](121)=1−FQ∥P(logγ)−γP[ıQ∥P(X)>logγ](122)=Eγ(Q∥P)−γEγ′(Q∥P)−γP[ıQ∥P(X)>logγ](123)=Eγ(Q∥P)−γEγ′(Q∥P)−γP[ıP∥Q(X)<−logγ](124)=Eγ(Q∥P)−γEγ′(Q∥P)−γFP∥Q(−logγ)

where (120) holds by switching *P* and *Q* in (46); (121) holds since Y∼Q; (122) holds by switching *P* and *Q* in (115) (correspondingly, also X∼P and Y∼Q are switched); (123) holds since ${ı}_{Q∥P}=-{ı}_{P∥Q}$; (124) holds by the assumption that $\frac{dP}{dQ}\;\left(X\right)$ has no probability masses, which implies that the sign < can be replaced with ≤ at the term $P[{ı}_{P∥Q}\left(X\right)<-\log\gamma ]$ in the right side of (123). Finally, (116) readily follows from (120)–(124), which implies that the set $A_{2}$ determines $F_{P∥Q}\left(\cdot \right)$ on (−∞,0).

Equalities (117) and (117) finally follows by letting γ↓1, respectively, on both sides of (115) and (116). ☐

A similar application of (107) yields an interplay between DeGroot statistical information and the relative information spectrum.

**Theorem** **4.** *Let X∼P, and let the random variable ${ı}_{P∥Q}\left(X\right)$ have no probability masses. Denote*

(125) $$\begin{array}{cc} & B_{1}:=\left\{I_{\omega }\left(P∥Q\right):\omega \in \left(0,\frac{1}{2}\right]\right\}, \end{array}$$

(126) $$\begin{array}{cc} & B_{2}:=\left\{I_{\omega }\left(P∥Q\right):\omega \in \left(\frac{1}{2},1\right)\right\}. \end{array}$$

*Then,*

- *$I_{\omega }\left(P∥Q\right)$ is a continuously differentiable function of ω on $\left(0,\frac{1}{2}\right)\cup \left(\frac{1}{2},1\right)$,*
  (127) $$\begin{array}{c} \lim_{\omega ↑\frac{1}{2}}\;I_{\omega }^{\prime }\left(P∥Q\right)-\lim_{\omega ↓\frac{1}{2}}\;I_{\omega }^{\prime }\left(P∥Q\right)=2, \end{array}$$
  *and $I_{\omega }^{\prime }\left(P∥Q\right)$ is, respectively, non-negative or non-positive on $\left(0,\frac{1}{2}\right)$ and $\left(\frac{1}{2},1\right)$;*
- *the sets $B_{1}$ and $B_{2}$ determine, respectively, the relative information spectrum $F_{P∥Q}\left(\cdot \right)$ on [0,∞) and (−∞,0);*
- *for $\omega \in \left(0,\frac{1}{2}\right)$*
  (128) $$\begin{array}{c} F_{P∥Q}\left(\log\frac{1-\omega }{\omega }\right)=1-I_{\omega }\left(P∥Q\right)-\left(1-\omega \right)\;I_{\omega }^{\prime }\left(P∥Q\right), \end{array}$$
  *for $\omega \in \left(\frac{1}{2},1\right)$*
  (129) $$\begin{array}{c} F_{P∥Q}\left(\log\frac{1-\omega }{\omega }\right)=-I_{\omega }\left(P∥Q\right)-\left(1-\omega \right)\;I_{\omega }^{\prime }\left(P∥Q\right), \end{array}$$
  *and*
  (130) $$\begin{array}{cc} F_{P∥Q}\left(0\right) & =-I_{\frac{1}{2}}\left(P∥Q\right)-\frac{1}{2}\lim_{\omega ↓\frac{1}{2}}\;I_{\omega }^{\prime }\left(P∥Q\right). \end{array}$$

**Remark** **8.** *By relaxing the condition in Theorems 3 and 4 where $\frac{dP}{dQ}\;\left(X\right)$ has no probability masses with X∼P, it follows from the proof of Theorem 3 that each one of the sets*

(131) $$\begin{array}{cc} & A:=A_{1}\cup A_{2}=\left\{\left(E_{\gamma }\left(P∥Q\right),E_{\gamma }\left(Q∥P\right)\right):\gamma \geq 1\right\}, \end{array}$$

(132) $$\begin{array}{cc} & B:=B_{1}\cup B_{2}=\left\{I_{\omega }\left(P∥Q\right):\omega \in \left(0,1\right)\right\} \end{array}$$

*determines $F_{P∥Q}\left(\cdot \right)$ at every point on R where this relative information spectrum is continuous. Note that, as a cumulative distribution function, $F_{P∥Q}\left(\cdot \right)$ is discontinuous at a countable number of points. Consequently, under the condition that f∈C is differentiable on (0,∞), the integral representations of $D_{f}\left(P∥Q\right)$ in Theorem 1 are not affected by the countable number of discontinuities for $F_{P∥Q}\left(\cdot \right)$.*

In view of Theorems 1, 3 and 4 and Remark 8, we get the following result.

**Corollary** **1.** *Let f∈C be a differentiable function on (0,∞), and let P≪≫Q be probability measures. Then, each one of the sets A and B in* (131) *and* (132)*, respectively, determines $D_{f}\left(P∥Q\right)$.*

**Remark** **9.** *Corollary 1 is supported by the integral representation of $D_{f}\left(P∥Q\right)$ in [3] Theorem 11, expressed as a function of the set of values in B, and its analogous representation in [22] Proposition 3 as a function of the set of values in A. More explicitly, [3] Theorem 11 states that if f∈C, then*

(133) $$\begin{array}{c} D_{f}\left(P∥Q\right)=\int_{0}^{1}I_{\omega }\left(P∥Q\right)\;d\Gamma_{f}\left(\omega \right) \end{array}$$

*where $\Gamma_{f}$ is a certain σ-finite measure defined on the Borel subsets of (0,1); it is also shown in [3] (80) that if f∈C is twice differentiable on (0,∞), then*

(134) $$\begin{array}{cc} D_{f}\left(P∥Q\right) & =\int_{0}^{1}I_{\omega }\left(P∥Q\right)\;\frac{1}{\omega^{3}}\;f^{\prime \prime }\left(\frac{\omega }{1-\omega }\right)\;d\omega . \end{array}$$

## 4. New f-Divergence Inequalities

Various approaches for the derivation of *f*-divergence inequalities were studied in the literature (see Section 1 for references). This section suggests a new approach, leading to a lower bound on an arbitrary *f*-divergence by means of the $E_{\gamma }$ divergence of an arbitrary order γ≥1 (see (45)) or the DeGroot statistical information (see (50)). This approach leads to generalizations of the Bretagnole-Huber inequality [58], whose generalizations are later motivated in this section. The utility of the *f*-divergence inequalities in this section is exemplified in the setup of Bayesian binary hypothesis testing.

In the following, we provide the first main result in this section for the derivation of new *f*-divergence inequalities by means of the $E_{\gamma }$ divergence. Generalizing the total variation distance, the $E_{\gamma }$ divergence in (45)–(47) is an *f*-divergence whose utility in information theory has been exemplified in [17] Chapter 3, [54],[57] p. 2314 and [69]; the properties of this measure were studied in [22] Section 7 and [54] Section 2.B.

**Theorem** **5.** *Let f∈C, and let $f^{*}\in C$ be the conjugate convex function as defined in (12). Let P and Q be probability measures. Then, for all γ∈[1,∞),*

(135) $$\begin{array}{c} D_{f}\left(P∥Q\right)\geq f^{*}\left(1+\frac{1}{\gamma }\;E_{\gamma }\left(P∥Q\right)\right)+f^{*}\left(\frac{1}{\gamma }\;\left(1-E_{\gamma }\left(P∥Q\right)\right)\right)-f^{*}\left(\frac{1}{\gamma }\right). \end{array}$$

**Proof.**  Let $p=\frac{dP}{d\mu }$ and $q=\frac{dQ}{d\mu }$ be the densities of *P* and *Q* with respect to a dominating measure μ(P,Q≪μ). Then, for an arbitrary a∈R,

(136)Df(P∥Q)=Df∗(Q∥P)(137)=∫pf∗qpdμ(138)=∫pf∗maxa,qp+f∗mina,qp−f∗(a)dμ(137)≥f∗∫pmaxa,qpdμ+f∗∫pmina,qpdμ−f∗(a)

where (139) follows from the convexity of $f^{*}$ and by invoking Jensen’s inequality.

Setting $a:=\frac{1}{\gamma }$ with γ∈[1,∞) gives

(140)∫pmaxa,qpdμ=∫maxpγ,qdμ(141)=∫qdμ+∫maxpγ−q,0dμ(142)=1+1γ∫qmaxpq−γ,0dμ(143)=1+1γEγ(P∥Q),

and

(143)∫pmina,qpdμ=∫pa+qp−maxa,qpdμ(145)=a+1−∫pmaxa,qpdμ(146)=1γ1−Eγ(P∥Q)

where (146) follows from (143) by setting $a:=\frac{1}{\gamma }$. Substituting (143) and (146) into the right side of (139) gives (135). ☐

An application of Theorem 5 gives the following lower bounds on the Hellinger and Rényi divergences with arbitrary positive orders, expressed as a function of the $E_{\gamma }$ divergence with an arbitrary order γ≥1.

**Corollary** **2.** *For all α>0 and γ≥1,*

(147) $$H_{\alpha }\left(P∥Q\right)\geq \{\begin{array}{cc} \frac{1}{\alpha -1}\left[{\left(1+\frac{1}{\gamma }\;E_{\gamma }\left(P∥Q\right)\right)}^{1-\alpha }+{\left(\frac{1-E_{\gamma }\left(P∥Q\right)}{\gamma }\right)}^{1-\alpha }-1-\gamma^{\alpha -1}\right], & \;\alpha \neq 1\\ -\log_{e}\left(\left(1+\frac{1}{\gamma }\;E_{\gamma }\left(P∥Q\right)\right)\left(1-E_{\gamma }\left(P∥Q\right)\right)\right), & \;\alpha =1, \end{array}$$

*and*

(148) $$D_{\alpha }\left(P∥Q\right)\geq \{\begin{array}{cc} \frac{1}{\alpha -1}\log\left({\left(1+\frac{1}{\gamma }\;E_{\gamma }\left(P∥Q\right)\right)}^{1-\alpha }+\gamma^{\alpha -1}\left[{\left(1-E_{\gamma }\left(P∥Q\right)\right)}^{1-\alpha }-1\right]\right), & \;\alpha \neq 1\\ -\log\left(\left(1+\frac{1}{\gamma }\;E_{\gamma }\left(P∥Q\right)\right)\left(1-E_{\gamma }\left(P∥Q\right)\right)\right), & \;\alpha =1. \end{array}$$

**Proof.**  Inequality (147), for α∈(0,1)∪(1,∞), follows from Theorem 5 and (22); for α=1, it holds in view of Theorem 5, and equalities (17) and (24). Inequality (148), for α∈(0,1)∪(1,∞), follows from (30) and (147); for α=1, it holds in view of (24), (147) and since $D_{1}\left(P∥Q\right)=D\left(P∥Q\right)$. ☐

Specialization of Corollary 2 for α=2 in (147) and α=1 in (148) gives the following result.

**Corollary** **3.** *For γ∈[1,∞), the following upper bounds on $E_{\gamma }$ divergence hold as a function of the relative entropy and $\chi^{2}$ divergence:*

(149) $$\begin{array}{cc} & E_{\gamma }\left(P∥Q\right)\leq \frac{1}{2}\left[1-\gamma +\sqrt{{\left(\gamma -1\right)}^{2}+\frac{4\gamma \;\chi^{2}\left(P∥Q\right)}{1+\gamma +\chi^{2}\left(P∥Q\right)}}\;\right], \end{array}$$

(150) $$\begin{array}{cc} & E_{\gamma }\left(P∥Q\right)\leq \frac{1}{2}\left[1-\gamma +\sqrt{{\left(\gamma -1\right)}^{2}+4\gamma \left(1-\exp(-D(P∥Q))\right)}\right]. \end{array}$$

**Remark** **10.** *From [4] (58),*

(151) $$\chi^{2}\left(P∥Q\right)\geq \{\begin{array}{cc} {\left|P-Q\right|}^{2}, & \;\left|P-Q|\in [0,1\right)\\ \frac{\left|P-Q\right|}{2-|P-Q|}, & \;\left|P-Q|\in [1,2\right) \end{array}$$

*is a tight lower bound on the chi-squared divergence as a function of the total variation distance. In view of* (49)*, we compare* (151) *with the specialized version of* (149) *when γ=1. The latter bound is expected to be looser than the tight bound in* (151)*, as a result of the use of Jensen’s inequality in the proof of Theorem 5; however, it is interesting to examine how much we loose in the tightness of this specialized bound with γ=1. From* (49)*, the substitution of γ=1 in* (149) *gives*

(152) $$\begin{array}{cc} \chi^{2}\left(P∥Q\right)\geq \frac{{2|P-Q|}^{2}}{{4-|P-Q|}^{2}}, & \;|P-Q|\in [0,2), \end{array}$$

*and, it can be easily verified that*

- *if |P−Q|∈[0,1), then the lower bound in the right side of* (152) *is at most twice smaller than the tight lower bound in the right side of* (151)*;*
- *if |P−Q|∈[1,2), then the lower bound in the right side of (152) is at most $\frac{3}{2}$ times smaller than the tight lower bound in the right side of* (151)*.*

**Remark** **11.** *Setting γ=1 in (150), and using (49), specializes to the Bretagnole-Huber inequality [58]:*

(153) $$\begin{array}{c} \left|P-Q\right|\leq 2\sqrt{1-\exp\left(-D(P∥Q)\right)}. \end{array}$$

Inequality (153) forms a counterpart to Pinsker’s inequality:

(154) $$\begin{array}{c} \frac{1}{2}{\left|P-Q\right|}^{2}\log e\leq D\left(P∥Q\right), \end{array}$$

proved by Csiszár [12] and Kullback [70], with Kemperman [71] independently a bit later. As upper bounds on the total variation distance, (154) outperforms (153) if D(P∥Q)≤1.594 nats, and (153) outperforms (154) for larger values of D(P∥Q).

**Remark** **12.** *In [59] (8), Vajda introduced a lower bound on the relative entropy as a function of the total variation distance:*

(155) $$\begin{array}{c} D\left(P∥Q\right)\geq \log\left(\frac{2+|P-Q|}{2-|P-Q|}\right)-\frac{2|P-Q|\;\log e}{2+|P-Q|},\;\left|P-Q\right|\in \left[0,2\right). \end{array}$$

*The lower bound in the right side of* (155) *is asymptotically tight in the sense that it tends to ∞ if |P−Q|↑2, and the difference between D(P∥Q) and this lower bound is everywhere upper bounded by $\frac{{2|P-Q|}^{3}}{{\left(2+|P-Q|\right)}^{2}}\leq 4$ (see [59] (9)). The Bretagnole-Huber inequality in* (153)*, on the other hand, is equivalent to*

(156) $$\begin{array}{c} D\left(P∥Q\right)\geq -\log\left(1-\frac{1}{4}{\left|P-Q\right|}^{2}\right),\;\left|P-Q\right|\in \left[0,2\right). \end{array}$$

*Although it can be verified numerically that the lower bound on the relative entropy in* (155) *is everywhere slightly tighter than the lower bound in* (156) *(for |P−Q|∈[0,2)), both lower bounds on D(P∥Q) are of the same asymptotic tightness in a sense that they both tend to ∞ as |P−Q|↑2 and their ratio tends to 1. Apart of their asymptotic tightness, the Bretagnole-Huber inequality in* (156) *is appealing since it provides a closed-form simple upper bound on |P−Q| as a function of D(P∥Q) (see* (153)*), whereas such a closed-form simple upper bound cannot be obtained from* (155)*. In fact, by the substitution $v:=-\frac{2-|P-Q|}{2+|P-Q|}$ and the exponentiation of both sides of* (155)*, we get the inequality $ve^{v}\geq -\frac{1}{e}\;\exp\left(-D(P∥Q)\right)$ whose solution is expressed by the Lambert W function [72]; it can be verified that* (155) *is equivalent to the following upper bound on the total variation distance as a function of the relative entropy:*

(157) $$\begin{array}{cc} & \left|P-Q\right|\leq \frac{2\left(1+W(z)\right)}{1-W(z)}, \end{array}$$

(158) $$\begin{array}{cc} & z:=-\frac{1}{e}\;\exp\left(-D(P∥Q)\right), \end{array}$$

*where W in the right side of* (157) *denotes the principal real branch of the Lambert W function. The difference between the upper bounds in* (153) *and* (157) *can be verified to be marginal if D(P∥Q) is large (e.g., if D(P∥Q)=4 nats, then the upper bounds on |P−Q| are respectively equal to 1.982 and 1.973), though the former upper bound in* (153) *is clearly more simple and amenable to analysis.*

*The Bretagnole-Huber inequality in* (153) *is proved to be useful in the context of lower bounding the minimax risk (see, e.g., [5] pp. 89–90, 94), and the problem of density estimation (see, e.g., [6] Section 1.6). The utility of this inequality motivates its generalization in this section (see Corollaries 2 and 3, and also see later Theorem 7 followed by Example 2).*

In [22] Section 7.C, Sason and Verdú generalized Pinsker’s inequality by providing an upper bound on the $E_{\gamma }$ divergence, for γ>1, as a function of the relative entropy. In view of (49) and the optimality of the constant in Pinsker’s inequality (154), it follows that the minimum achievable D(P∥Q) is quadratic in $E_{1}\left(P∥Q\right)$ for small values of $E_{1}\left(P∥Q\right)$. It has been proved in [22] Section 7.C that this situation ceases to be the case for γ>1, in which case it is possible to upper bound $E_{\gamma }\left(P∥Q\right)$ as a constant times D(P∥Q) where this constant tends to infinity as we let γ↓1. We next cite the result in [22] Theorem 30, extending (154) by means of the $E_{\gamma }$ divergence for γ>1, and compare it numerically to the bound in (150).

**Theorem** **6.** *([22] Theorem 30) For every γ>1,*

(159) $$\begin{array}{c} \sup\frac{E_{\gamma }\left(P∥Q\right)}{D(P∥Q)}=c_{\gamma } \end{array}$$

*where the supremum is over P≪Q,P≠Q, and $c_{\gamma }$ is a universal function (independent of (P,Q)), given by*

(160) $$\begin{array}{cc} & c_{\gamma }=\frac{t_{\gamma }-\gamma }{t_{\gamma }\;\log t_{\gamma }+\left(1-t_{\gamma }\right)\;\log e}, \end{array}$$

(161) $$\begin{array}{cc} & t_{\gamma }=-\gamma \;W_{-1}\left(-\frac{1}{\gamma }\;e^{-\frac{1}{\gamma }}\right) \end{array}$$

*where $W_{-1}$ in (161) denotes the secondary real branch of the Lambert W function [72].*

As an immediate consequence of (159), it follows that

(162) $$\begin{array}{c} E_{\gamma }\left(P∥Q\right)\leq c_{\gamma }D\left(P∥Q\right), \end{array}$$

which forms a straight-line bound on the $E_{\gamma }$ divergence as a function of the relative entropy for γ>1. Similarly to the comparison of the Bretagnole-Huber inequality (153) and Pinsker’s inequality (154), we exemplify numerically that the extension of Pinsker’s inequality to the $E_{\gamma }$ divergence in (162) forms a counterpart to the generalized version of the Bretagnole-Huber inequality in (150).

**Figure 1:**
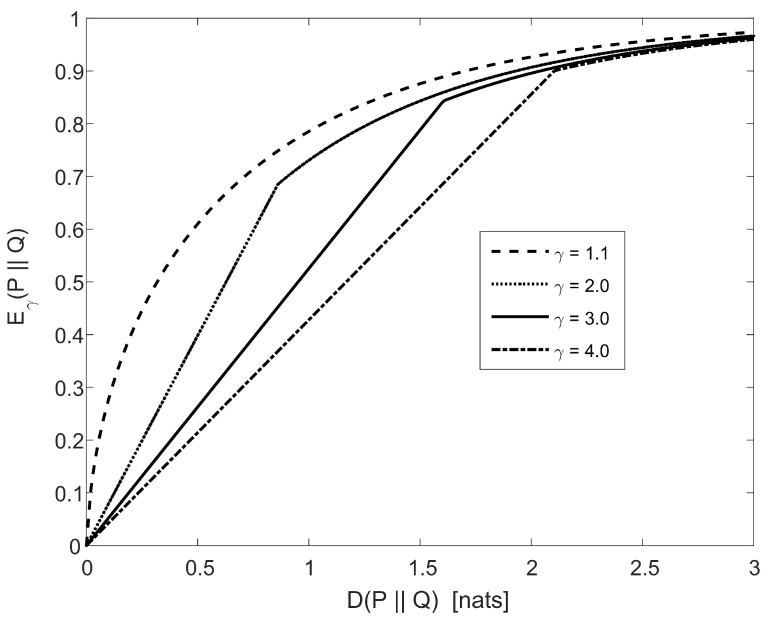
Upper bounds on the $E_{\gamma }$ divergence, for γ>1, as a function of the relative entropy (the curvy and straight lines follow from (150) and (162), respectively).

Figure 1 plots an upper bound on the $E_{\gamma }$ divergence, for γ∈{1.1,2.0,3.0,4.0}, as a function of the relative entropy (or, alternatively, a lower bound on the relative entropy as a function of the $E_{\gamma }$ divergence). The upper bound on $E_{\gamma }\left(P∥Q\right)$ for γ>1, as a function of D(P∥Q), is composed of the following two components:

- the straight-line bound, which refers to the right side of (162), is tighter than the bound in the right side of (150) if the relative entropy is below a certain value that is denoted by d(γ) in nats (it depends on γ);
- the curvy line, which refers to the bound in the right side of (150), is tighter than the straight-line bound in the right side of (162) for larger values of the relative entropy.

It is supported by Figure 1 that d:(1,∞)↦(0,∞) is positive and monotonically increasing, and $\lim_{\gamma ↓1}\;d\left(\gamma \right)=0$; e.g., it can be verified that d(1.1)≈0.02, d(2)≈0.86, d(3)≈1.61, and d(4)≈2.10 (see Figure 1).

### Bayesian Binary Hypothesis Testing

The DeGroot statistical information [16] has the following meaning: consider two hypotheses $H_{0}$ and $H_{1}$, and let $P[H_{0}]=\omega$ and $P[H_{1}]=1-\omega$ with ω∈(0,1). Let *P* and *Q* be probability measures, and consider an observation *Y* where $Y|H_{0}∼P$, and $Y|H_{1}∼Q$. Suppose that one wishes to decide which hypothesis is more likely given the observation *Y*. The operational meaning of the DeGroot statistical information, denoted by $I_{\omega }\left(P∥Q\right)$, is that this measure is equal to the minimal difference between the *a-priori* error probability (without side information) and *a posteriori* error probability (given the observation *Y*). This measure was later identified as an *f*-divergence by Liese and Vajda [3] (see (50) here).

**Theorem** **7.** *The DeGroot statistical information satisfies the following upper bound as a function of the chi-squared divergence:*

(163) $$I_{\omega }\left(P∥Q\right)\leq \{\begin{array}{cc} \omega -\frac{1}{2}+\sqrt{\frac{1}{4}-\frac{\omega (1-\omega )}{1+\omega \;\chi^{2}\left(P∥Q\right)}}\;, & \;\omega \in \left(0,\frac{1}{2}\right],\\ \frac{1}{2}-\omega +\sqrt{\frac{1}{4}-\frac{\omega (1-\omega )}{1+\omega \;\chi^{2}\left(Q∥P\right)}}\;, & \;\omega \in \left(\frac{1}{2},1\right), \end{array}$$

*and the following bounds as a function of the relative entropy:*

*(1)*  (164) $$I_{\omega }\left(P∥Q\right)\leq \{\begin{array}{cc} \omega \;c_{\frac{1-\omega }{\omega }}\;D\left(P∥Q\right)\;, & \;\omega \in \left(0,\frac{1}{2}\right),\\ \sqrt{\frac{1}{8\log e}\;\min\left\{D(P∥Q),D(Q∥P)\right\}}\;, & \;\omega =\frac{1}{2},\\ \left(1-\omega \right)\;c_{\frac{\omega }{1-\omega }}\;D\left(Q∥P\right)\;, & \;\omega \in \left(\frac{1}{2},1\right), \end{array}$$
  *where $c_{\gamma }$ for γ>1 is introduced in* (160)*;*
*(2)*  (165) $$I_{\omega }\left(P∥Q\right)\leq \{\begin{array}{cc} \omega -\frac{1}{2}+\sqrt{\frac{1}{4}-\omega \left(1-\omega \right)\;\exp\left(-D(P∥Q)\right)}\;, & \;\omega \in \left(0,\frac{1}{2}\right],\\ \frac{1}{2}-\omega +\sqrt{\frac{1}{4}-\omega \left(1-\omega \right)\;\exp\left(-D(Q∥P)\right)}\;, & \;\omega \in \left(\frac{1}{2},1\right). \end{array}$$

**Proof.**  The first bound in (163) holds by combining (53) and (149); the second bound in (164) follows from (162) and (53) for $\omega \in \left(0,\frac{1}{2}\right)\cup \left(\frac{1}{2},1\right)$, and it follows from (52) and (154) when $\omega =\frac{1}{2}$; finally, the third bound in (165) follows from (150) and (53). ☐

**Remark** **13.** *The bound in* (164) *forms an extension of Pinsker’s inequality* (154) *when $\omega \neq \frac{1}{2}$ (i.e., in the asymmetric case where the hypotheses $H_{0}$ and $H_{1}$ are not equally probable). Furthermore, in view of* (52)*, the bound in* (165) *is specialized to the Bretagnole-Huber inequality in* (153) *by letting* $\omega =\frac{1}{2}$.

**Remark** **14.** *Numerical evidence shows that none of the bounds in* (163)–(165) *supersedes the others.*

**Remark** **15.** *The upper bounds on $I_{\omega }\left(P_{\mu }∥P_{\lambda }\right)$ in* (163) *and* (165) *are asymptotically tight when we let D(P∥Q) and D(Q∥P) tend to infinity. To verify this, first note that (see [23] Theorem 5)*

(166) $$\begin{array}{cc} D(P∥Q) & \leq \log\left(1+\chi^{2}\left(P∥Q\right)\right), \end{array}$$

*which implies that also $\chi^{2}\left(P∥Q\right)$ and $\chi^{2}\left(Q∥P\right)$ tend to infinity. In this case, it can be readily verified that the bounds in* (163) *and* (165) *are specialized to $I_{\omega }\left(P∥Q\right)\leq \min\left\{\omega ,1-\omega \right\}$; this upper bound, which is equal to the* a-priori *error probability, is also equal to the DeGroot statistical information since the a-posterior error probability tends to zero in the considered extreme case where P and Q are sufficiently far from each other, so that $H_{0}$ and $H_{1}$ are easily distinguishable in high probability when the observation Y is available.*

**Remark** **16.** *Due to the one-to-one correspondence between the $E_{\gamma }$ divergence and DeGroot statistical information in* (53)*, which shows that the two measures are related by a multiplicative scaling factor, the numerical results shown in Figure 1 also apply to the bounds in* (164) *and* (165)*; i.e., for $\omega \neq \frac{1}{2}$, the first bound in* (164) *is tighter than the second bound in* (165) *for small values of the relative entropy, whereas* (165) *becomes tighter than* (164) *for larger values of the relative entropy.*

**Corollary** **4.** *Let f∈C, and let $f^{*}\in C$ be as defined in* (12)*. Then,*

*(1)*  *for $w\in (0,\frac{1}{2}]$,*
  (167) $$\begin{array}{c} D_{f}\left(P∥Q\right)\geq f^{*}\left(1+\frac{I_{w}\left(P∥Q\right)}{1-w}\right)+f^{*}\left(\frac{w-I_{w}\left(P∥Q\right)}{1-w}\right)-f^{*}\left(\frac{w}{1-w}\right); \end{array}$$
*(2)*  *for $w\in \left(\frac{1}{2},1\right)$,*
  (168) $$\begin{array}{c} D_{f}\left(P∥Q\right)\geq f^{*}\left(1+\frac{I_{w}\left(Q∥P\right)}{w}\right)+f^{*}\left(\frac{1-w-I_{w}\left(Q∥P\right)}{w}\right)-f^{*}\left(\frac{1-w}{w}\right). \end{array}$$

**Proof.**  Inequalities (167) and (168) follow by combining (135) and (53). ☐

We end this section by exemplifying the utility of the bounds in Theorem 7.

**Example** **2.** *Let $P[H_{0}]=\omega$ and $P[H_{1}]=1-\omega$ with ω∈(0,1), and assume that the observation Y given that the hypothesis is $H_{0}$ or $H_{1}$ is Poisson distributed with the positive parameter μ or λ, respectively:*

(169) $$\begin{array}{c} Y|H_{0}∼P_{\mu }, \end{array}$$

(170) $$\begin{array}{c} Y|H_{1}∼P_{\lambda } \end{array}$$

*where*

(171) $$\begin{array}{c} P_{\lambda }\left[k\right]=\frac{e^{-\lambda }\lambda^{k}}{k!},\;k\in \left\{0,1,\ldots \right\}. \end{array}$$

*Without any loss of generality, let $\omega \in \left(0,\frac{1}{2}\right]$. The bounds on the DeGroot statistical information $I_{\omega }\left(P_{\mu }∥P_{\lambda }\right)$ in Theorem 7 can be expressed in a closed form by relying on the following identities:*

(172) $$\begin{array}{c} D\left(P_{\mu }∥P_{\lambda }\right)=\mu \log\left(\frac{\mu }{\lambda }\right)+\left(\lambda -\mu \right)\log e, \end{array}$$

(173) $$\begin{array}{c} \chi^{2}\left(P_{\mu }∥P_{\lambda }\right)=e^{\frac{{\left(\mu -\lambda \right)}^{2}}{\lambda }}-1. \end{array}$$

*In this example, we compare the simple closed-form bounds on $I_{\omega }\left(P_{\mu }∥P_{\lambda }\right)$ in* (163)–(165) *with its exact value*

(174) $$\begin{array}{cc} I_{\omega }\left(P_{\mu }∥P_{\lambda }\right) & =\min\left\{\omega ,1-\omega \right\}-\sum_{k=0}^{\infty }\min\left\{\omega P_{\mu }\left[k\right],\left(1-\omega \right)P_{\lambda }\left[k\right]\right\}. \end{array}$$

*To simplify the right side of* (174)*, let μ>λ, and define*

(175) $$\begin{array}{c} k_{0}=k_{0}\left(\lambda ,\mu ,\omega \right):=\left\lfloor \frac{\mu -\lambda +\ln\frac{1-\omega }{\omega }}{\ln\frac{\mu }{\lambda }}\right\rfloor , \end{array}$$

*where for x∈R, ⌊x⌋ denotes the largest integer that is smaller than or equal to x. It can be verified that*

(176) $$\left\{ \begin{array}{cc} \omega P_{\mu }\left[k\right]\leq \left(1-\omega \right)P_{\lambda }\left[k\right], & \;fork\leq k_{0}\\ \omega P_{\mu }\left[k\right]>\left(1-\omega \right)P_{\lambda }\left[k\right], & \;fork>k_{0}. \end{array}\right.$$

*Hence, from* (174)–(176)*,*

(177)Iω(Pμ∥Pλ)=min{ω,1−ω}−ω∑k=0k0Pμ[k]−(1−ω)∑k=k0+1∞Pλ[k](178)=min{ω,1−ω}−ω∑k=0k0Pμ[k]−(1−ω)1−∑k=0k0Pλ[k].

*To exemplify the utility of the bounds in Theorem 7, suppose that μ and λ are close, and we wish to obtain a guarantee on how small $I_{\omega }\left(P_{\mu }∥P_{\lambda }\right)$ is. For example, let λ=99, μ=101, and $\omega =\frac{1}{10}$. The upper bounds on $I_{\omega }\left(P_{\mu }∥P_{\lambda }\right)$ in* (163)–(165) *are, respectively, equal to $4.6\cdot 10^{-4}$, $5.8\cdot 10^{-4}$ and $2.2\cdot 10^{-3}$; we therefore get an informative guarantee by easily calculable bounds. The exact value of $I_{\omega }\left(P_{\mu }∥P_{\lambda }\right)$ is, on the other hand, hard to compute since $k_{0}=209$ (see* (175)*), and the calculation of the right side of* (178) *appears to be sensitive to the selected parameters in this setting.*

## 5. Local Behavior of *f*-Divergences

This section studies the local behavior of *f*-divergences; the starting point relies on [56] Section 3 which studies the asymptotic properties of *f*-divergences. The reader is also referred to a related study in [22] Section 4.F.

**Lemma** **4.** *Let*

- *$\left\{P_{n}\right\}$ be a sequence of probability measures on a measurable space (A,F);*
- *the sequence $\left\{P_{n}\right\}$ converge to a probability measure Q in the sense that*
  (179) $$\begin{array}{c} \lim_{n\to \infty }\mathrm{ess}\;\sup\frac{dP_{n}}{dQ}\;\left(Y\right)=1,\;Y∼Q \end{array}$$
  *where $P_{n}\ll Q$ for all sufficiently large n;*
- *f,g∈C have continuous second derivatives at 1 and $g^{\prime \prime }\left(1\right)>0$.*

*Then*

(180) $$\begin{array}{c} \lim_{n\to \infty }\frac{D_{f}\left(P_{n}∥Q\right)}{D_{g}\left(P_{n}∥Q\right)}=\frac{f^{\prime \prime }\left(1\right)}{g^{\prime \prime }\left(1\right)}. \end{array}$$

**Proof.**  The result in (180) follows from [56] Theorem 3, even without the additional restriction in [56] Section 3 which would require that the second derivatives of *f* and *g* are locally Lipschitz at a neighborhood of 1. More explicitly, in view of the analysis in [56] p. 1863, we get by relaxing the latter restriction that (cf. [56] (31))

(181) $$\begin{array}{c} \left|D_{f}\left(P_{n}∥Q\right)-\frac{1}{2}\;f^{\prime \prime }\left(1\right)\;\chi^{2}\left(P_{n}∥Q\right)\right|\leq \frac{1}{2}\;\underset{y\in [1-\varepsilon_{n},1+\varepsilon_{n}]}{\sup}\left|f^{\prime \prime }\left(y\right)-f^{\prime \prime }\left(1\right)\right|\;\chi^{2}\left(P_{n}∥Q\right), \end{array}$$

where $\varepsilon_{n}↓0$ as we let n→∞, and also

(182) $$\begin{array}{c} \lim_{n\to \infty }\chi^{2}\left(P_{n}∥Q\right)=0. \end{array}$$

By our assumption, due to the continuity of $f^{\prime \prime }$ and $g^{\prime \prime }$ at 1, it follows from (181) and (182) that

(183) $$\begin{array}{c} \lim_{n\to \infty }\frac{D_{f}\left(P_{n}∥Q\right)}{\chi^{2}\left(P_{n}∥Q\right)}=\frac{1}{2}\;f^{\prime \prime }\left(1\right), \end{array}$$

(184) $$\begin{array}{c} \lim_{n\to \infty }\frac{D_{g}\left(P_{n}∥Q\right)}{\chi^{2}\left(P_{n}∥Q\right)}=\frac{1}{2}\;g^{\prime \prime }\left(1\right), \end{array}$$

which yields (180) (recall that, by assumption, $g^{\prime \prime }\left(1\right)>0$). ☐

**Remark** **17.** *Since f and g in Lemma 4 are assumed to have continuous second derivatives at 1, the left and right derivatives of the weight function $w_{f}$ in* (79) *at 1 satisfy, in view of Remark 3,*

(185) $$\begin{array}{c} w_{f}^{\prime }\left(1^{+}\right)=-w_{f}^{\prime }\left(1^{-}\right)=f^{\prime \prime }\left(1\right). \end{array}$$

*Hence, the limit in the right side of* (180) *is equal to $\frac{w_{f}^{\prime }\left(1^{+}\right)}{w_{g}^{\prime }\left(1^{+}\right)}$ or also to $\frac{w_{f}^{\prime }\left(1^{-}\right)}{w_{g}^{\prime }\left(1^{-}\right)}$.*

**Lemma** **5.** (186) $$\begin{array}{c} \chi^{2}\left(\lambda P+\left(1-\lambda \right)Q\;∥\;Q\right)=\lambda^{2}\;\chi^{2}\left(P∥Q\right),\;\forall \;\lambda \in \left[0,1\right]. \end{array}$$

**Proof.**  Let $p=\frac{dP}{d\mu }$ and $q=\frac{dQ}{d\mu }$ be the densities of *P* and *Q* with respect to an arbitrary probability measure μ such that P,Q≪μ. Then,

(187)χ2(λP+(1−λ)Q∥Q)=∫(λp+(1−λ)q)−q2qdμ(188)=λ2∫(p−q)2qdμ(189)=λ2χ2(P∥Q).

☐

**Remark** **18.** *The result in Lemma 5, for the chi-squared divergence, is generalized to the identity*

(190) $$\begin{array}{c} \chi^{s}\left(\lambda P+\left(1-\lambda \right)Q\;∥\;Q\right)=\lambda^{s}\;\chi^{s}\left(P∥Q\right),\;\forall \;\lambda \in \left[0,1\right], \end{array}$$

*for all s≥1 (see* (33)*). The special case of s=2 is required in the continuation of this section.*

**Remark** **19.** *The result in Lemma 5 can be generalized as follows: let P,Q,R be probability measures, and λ∈[0,1]. Let P,Q,R≪μ for an arbitrary probability measure μ, and $p:=\frac{dP}{d\mu }$, $q:=\frac{dQ}{d\mu }$, and $r:=\frac{dR}{d\mu }$ be the corresponding densities with respect to μ. Calculation shows that*

(191) $$\begin{array}{cc} \chi^{2}\left(\lambda P+\left(1-\lambda \right)Q\;∥\;R\right)-\chi^{2}\left(Q∥R\right) & =c\lambda +\left[\chi^{2}\left(P∥R\right)-\chi^{2}\left(Q∥R\right)-c\right]\lambda^{2} \end{array}$$

*with*

(192) $$\begin{array}{cc} c & :=\int \frac{(p-q)q}{r}\;d\mu . \end{array}$$

*If Q=R, then c=0 in* (192)*, and* (191) *is specialized to* (186)*. However, if Q≠R, then c may be non-zero. This shows that, for small λ∈[0,1], the left side of* (191) *scales linearly in λ if c≠0, and it has a quadratic scaling in λ if c=0 and $\chi^{2}\left(P∥R\right)\neq \chi^{2}\left(Q∥R\right)$ (e.g., if Q=R, as in Lemma 5). The identity in (191) yields*

(193) $$\begin{array}{c} \frac{d}{d\lambda }\;\chi^{2}\left(\lambda P+\left(1-\lambda \right)Q\;∥\;R\right)\;{|}_{\lambda =0}=\lim_{\lambda ↓0}\;\frac{\chi^{2}\left(\lambda P+\left(1-\lambda \right)Q\;∥\;R\right)-\chi^{2}\left(Q∥R\right)}{\lambda }=c. \end{array}$$

We next state the main result in this section.

**Theorem** **8.** *Let*

- *P and Q be probability measures defined on a measurable space (A,F), Y∼Q, and suppose that*
  (194) $$\begin{array}{c} \mathrm{ess}\;\sup\frac{dP}{dQ}\;\left(Y\right)<\infty ; \end{array}$$
- *f∈C, and $f^{\prime \prime }$ be continuous at 1.*

*Then,*

(195)limλ↓01λ2Df(λP+(1−λ)Q∥Q)=limλ↓01λ2Df(Q∥λP+(1−λ)Q)(196)=12f″(1)χ2(P∥Q).

**Proof.**  Let ${\left\{\lambda_{n}\right\}}_{n\in N}$ be a sequence in [0,1], which tends to zero. Define the sequence of probability measures

(197) $$\begin{array}{c} R_{n}:=\lambda_{n}P+\left(1-\lambda_{n}\right)Q,\;n\in N. \end{array}$$

Note that P≪Q implies that $R_{n}\ll Q$ for all n∈N. Since

(198) $$\begin{array}{c} \frac{dR_{n}}{dQ}=\lambda_{n}\;\frac{dP}{dQ}+\left(1-\lambda_{n}\right), \end{array}$$

it follows from (194) that

(199) $$\begin{array}{c} \lim_{n\to \infty }\mathrm{ess}\;\sup\frac{dR_{n}}{dQ}\;\left(Y\right)=1. \end{array}$$

Consequently, (183) implies that

(200) $$\begin{array}{c} \lim_{n\to \infty }\frac{D_{f}\left(R_{n}∥Q\right)}{\chi^{2}\left(R_{n}∥Q\right)}=\frac{1}{2}\;f^{\prime \prime }\left(1\right) \end{array}$$

where $\left\{\lambda_{n}\right\}$ in (197) is an arbitrary sequence which tends to zero. Hence, it follows from (197) and (200) that

(201) $$\begin{array}{c} \lim_{\lambda ↓0}\frac{D_{f}\left(\lambda P+\left(1-\lambda \right)Q\;∥\;Q\right)}{\chi^{2}\left(\lambda P+\left(1-\lambda \right)Q\;∥\;Q\right)}=\frac{1}{2}\;f^{\prime \prime }\left(1\right), \end{array}$$

and, by combining (186) and (201), we get

(202) $$\begin{array}{c} \lim_{\lambda ↓0}\frac{1}{\lambda^{2}}\;D_{f}\left(\lambda P+\left(1-\lambda \right)Q\;∥\;Q\right)=\frac{1}{2}\;f^{\prime \prime }\left(1\right)\;\chi^{2}\left(P∥Q\right). \end{array}$$

We next prove the result for the limit in the right side of (195). Let $f^{*}:\left(0,\infty \right)\mapsto R$ be the conjugate function of *f*, which is given in (12). By the assumption that *f* has a second continuous derivative, so is $f^{*}$ and it is easy to verify that the second derivatives of *f* and $f^{*}$ coincide at 1. Hence, from (13) and (202),

(203)limλ↓01λ2Df(Q∥λP+(1−λ)Q)=limλ↓01λ2Df∗(λP+(1−λ)Q∥Q)(204)=12f″(1)χ2(P∥Q).

☐

**Remark** **20.** *Although an f-divergence is in general not symmetric, in the sense that the equality $D_{f}\left(P∥Q\right)=D_{f}\left(Q∥P\right)$ does not necessarily hold for all pairs of probability measures (P,Q), the reason for the equality in* (195) *stems from the fact that the second derivatives of f and $f^{*}$ coincide at 1 when f is twice differentiable.*

**Remark** **21.** *Under the conditions in Theorem 8, it follows from* (196) *that*

(205) $$\begin{array}{cc} & \frac{d}{d\lambda }\;D_{f}\left(\lambda P+\left(1-\lambda \right)Q\;∥\;Q\right)\;{|}_{\lambda =0}=\lim_{\lambda ↓0}\frac{1}{\lambda }\;D_{f}\left(\lambda P+\left(1-\lambda \right)Q\;∥\;Q\right)=0, \end{array}$$

(206) $$\begin{array}{cc} & \lim_{\lambda ↓0}\;\frac{d^{2}}{d\lambda^{2}}\;D_{f}\left(\lambda P+\left(1-\lambda \right)Q\;∥\;Q\right)=2\;\lim_{\lambda ↓0}\frac{1}{\lambda^{2}}\;D_{f}\left(\lambda P+\left(1-\lambda \right)Q\;∥\;Q\right)=f^{\prime \prime }\left(1\right)\;\chi^{2}\left(P∥Q\right) \end{array}$$

*where* (206) *relies on L’Hôpital’s rule. The convexity of $D_{f}\left(P∥Q\right)$ in (P,Q) also implies that, for all λ∈[0,1],*

(207) $$\begin{array}{c} D_{f}\left(\lambda P+\left(1-\lambda \right)Q\;∥\;Q\right)\leq \lambda D_{f}\left(P∥Q\right). \end{array}$$

The following result refers to the local behavior of Rényi divergences of an arbitrary non-negative order.

**Corollary** **5.** *Under the condition in* (194)*, for every α∈[0,∞],*

(208)limλ↓01λ2Dα(λP+(1−λ)Q∥Q)=limλ↓01λ2Dα(Q∥λP+(1−λ)Q)(209)=12αχ2(P∥Q)loge.

**Proof.**  Let α∈(0,1)∪(1,∞). In view of (23) and Theorem 8, it follows that the local behavior of the Hellinger divergence of order α satisfies

(210)limλ↓01λ2Hα(λP+(1−λ)Q∥Q)=limλ↓01λ2Hα(Q∥λP+(1−λ)Q)(211)=12αχ2(P∥Q).

The result now follows from (30), which implies that

(212)limλ↓0Dα(λP+(1−λ)Q∥Q)Hα(λP+(1−λ)Q∥Q)=limλ↓0Dα(Q∥λP+(1−λ)Q)Hα(Q∥λP+(1−λ)Q)(213)=1α−1limu→0log1+(α−1)uu(214)=loge.

The result in (208) and (209), for α∈(0,1)∪(1,∞), follows by combining the equalities in (210)–(214).

Finally, the result in (208) and (209) for α∈{0,1,∞} follows from its validity for all α∈(0,1)∪(1,∞), and also due to the property where $D_{\alpha }\left(\cdot ∥\cdot \right)$ is monotonically increasing in α (see [73] Theorem 3). ☐

## Appendix A. Proof of Theorem 2

We prove in the following the integral representations of *f*-divergences and related measures in Theorem 2.

(1) Relative entropy: The function f∈C in (18) yields the following weight function in (79):
  (A1) $$\begin{array}{c} w_{f}\left(\beta \right)=\left(\frac{1}{\beta }-\frac{1}{\beta^{2}}\right)\left(1\{\beta \geq 1\}-1\{0<\beta <1\}\right)\;\log e,\;\beta >0. \end{array}$$
  Consequently, setting c:=loge in (82) yields
  (A2) $$\begin{array}{c} {\tilde{w}}_{f,c}\left(\beta \right)=\frac{1}{\beta }\left(1\{\beta \geq 1\}-1\{0<\beta <1\}\right)\log e, \end{array}$$
  for β>0. Equality (98) follows from the substitution of (A2) into the right side of (83).
(2) Hellinger divergence: In view of (22), for α∈(0,1)∪(1,∞), the weight function $w_{f_{\alpha }}:\left(0,\infty \right)\mapsto \left[0,\infty \right)$ in (79) which corresponds to $f_{\alpha }:\left(0,\infty \right)\mapsto R$ in (23) can be verified to be equal to
  (A3) $$\begin{array}{c} w_{f_{\alpha }}\left(\beta \right)=\left(\beta^{\alpha -2}-\frac{1}{\beta^{2}}\right)\left(1\{\beta \geq 1\}-1\{0<\beta <1\}\right) \end{array}$$
  for β>0. In order to simplify the integral representation of the Hellinger divergence $H_{\alpha }\left(P∥Q\right)$, we apply Theorem 1-(1). From (A3), setting c:=1 in (82) implies that ${\tilde{w}}_{f_{\alpha },1}:\left(0,\infty \right)\to R$ is given by
  (A4) $$\begin{array}{c} {\tilde{w}}_{f_{\alpha },1}\left(\beta \right)=\beta^{\alpha -2}\left(1\{\beta \geq 1\}-1\{0<\beta <1\}\right) \end{array}$$
  for β>0. Hence, substituting (80) and (A4) into (83) yields
  (A5) $$\begin{array}{c} H_{\alpha }\left(P∥Q\right)=\int_{1}^{\infty }\beta^{\alpha -2}\;\left(1-F_{P∥Q}\left(\log\beta \right)\right)\;d\beta -\int_{0}^{1}\beta^{\alpha -2}\;F_{P∥Q}\left(\log\beta \right)\;d\beta . \end{array}$$
  For α>1, (A5) yields
  (A6)Hα(P∥Q)=∫0∞βα−21−FP∥Q(logβ)dβ−∫01βα−2dβ(A7)=∫0∞βα−21−FP∥Q(logβ)dβ−1α−1,
  and, for α∈(0,1), (A5) yields
  (A8)Hα(P∥Q)=∫1∞βα−2dβ−∫0∞βα−2FP∥Q(logβ)dβ(A9)=11−α−∫0∞βα−2FP∥Q(logβ)dβ.
  This proves (99). We next consider the following special cases:
  - In view of (25), equality (100) readily follows from (99) with α=2.
  - In view of (28), equality (101) readily follows from (99) with $\alpha =\frac{1}{2}$.
  - In view of (29), equality (102) readily follows from (101).
(3) Rényi divergence: In view of the one-to-one correspondence in (30) between the Rényi divergence and the Hellinger divergence of the same order, (103) readily follows from (99).
(4) $\chi^{s}$ divergence with s≥1: We first consider the case where s>1. From (33), the function $f_{s}:\left(0,\infty \right)\mapsto R$ in (32) is differentiable and $f_{s}^{\prime }\left(1\right)=0$. Hence, the respective weight function $w_{f_{s}}:\left(0,\infty \right)\mapsto \left(0,\infty \right)$ can be verified from (79) to be given by
  (A10) $$\begin{array}{c} w_{f_{s}}\left(\beta \right)=\frac{1}{\beta }\left(s-1+\frac{1}{\beta }\right){\left|\beta -1\right|}^{s-1},\;\beta >0. \end{array}$$
  The result in (104), for s>1, follows readily from (33), (80), (81) and (A10).
  We next prove (104) with s=1. In view of (32), (34), (35) and the dominated convergence theorem,
  (A11)|P−Q|=lims↓1χs(P∥Q)(A12)=∫1∞1−FP∥Q(logβ)β2dβ+∫01FP∥Q(logβ)β2dβ.
  This extends (104) for all s≥1, although $f_{1}\left(t\right)=\left|t-1\right|$ for t>0 is not differentiable at 1. For s=1, in view of (95), the integral representation in the right side of (A12) can be simplified to (105) and (106).
(5) DeGroot statistical information: In view of (50)–(51), since the function $\phi_{w}:\left(0,\infty \right)\mapsto R$ is not differentiable at the point $\frac{1-\omega }{\omega }\in \left(0,\infty \right)$ for ω∈(0,1), Theorem 1 cannot be applied directly to get an integral representation of the DeGroot statistical information. To that end, for $\left(\omega ,\alpha \right)\in {\left(0,1\right)}^{2}$, consider the family of convex functions $f_{\omega ,\alpha }:\left(0,\infty \right)\mapsto R$ given by (see [3] (55))
  (A13) $$\begin{array}{c} f_{\omega ,\alpha }\left(t\right)=\frac{1}{1-\alpha }\left({\left[{\left(\omega t\right)}^{\frac{1}{\alpha }}+{\left(1-\omega \right)}^{\frac{1}{\alpha }}\right]}^{\alpha }-{\left[\omega^{\frac{1}{\alpha }}+{\left(1-\omega \right)}^{\frac{1}{\alpha }}\right]}^{\alpha }\right), \end{array}$$
  for t>0. These differentiable functions also satisfy
  (A14) $$\begin{array}{c} \lim_{\alpha ↓0}f_{\omega ,\alpha }\left(t\right)=\phi_{w}\left(t\right), \end{array}$$
  which holds due to the identities
  (A15) $$\begin{array}{cc} & \lim_{\alpha ↓0}{\left(a^{\frac{1}{\alpha }}+b^{\frac{1}{\alpha }}\right)}^{\alpha }=\max\left\{a,b\right\},\;\;\;a,b\geq 0; \end{array}$$
  (A16) $$\begin{array}{cc} & \min\{a,b\}=a+b-\max\{a,b\},\;a,b\in R. \end{array}$$
  The application of Theorem 1-(1) to the set of functions $f_{\omega ,\alpha }\in C$ with
  (A17) $$\begin{array}{c} c:=\frac{{\left(1-\omega \right)}^{\frac{1}{\alpha }}}{\alpha -1}{\left[\omega^{\frac{1}{\alpha }}+{\left(1-\omega \right)}^{\frac{1}{\alpha }}\right]}^{\alpha -1} \end{array}$$
  yields
  (A18) $$\begin{array}{c} {\tilde{w}}_{f_{\omega ,\alpha },\;c}\left(\beta \right)=\frac{1-\omega }{1-\alpha }\;\frac{1}{\beta^{2}}{\left[1+{\left(\frac{\omega \beta }{1-\omega }\right)}^{\frac{1}{\alpha }}\right]}^{\alpha -1}\;\left[1\{0<\beta <1\}-1\{\beta \geq 1\}\right], \end{array}$$
  for β>0, and
  (A19) $$\begin{array}{c} D_{f_{\omega ,\alpha }}\left(P∥Q\right)=\int_{0}^{\infty }{\tilde{w}}_{f_{\omega ,\alpha },\;c}\left(\beta \right)\;G_{P∥Q}\left(\beta \right)\;d\beta \end{array}$$
  with $G_{P∥Q}\left(\cdot \right)$ as defined in (80), and $\left(\omega ,\alpha \right)\in {\left(0,1\right)}^{2}$. From (A15) and (A18), it follows that
  (A20) $$\begin{array}{c} \lim_{\alpha ↓0}\;{\tilde{w}}_{f_{\omega ,\alpha },\;c}\left(\beta \right)=\frac{1-\omega }{\beta^{2}}\;\left[1\{0<\beta <1\}-1\{\beta \geq 1\}\right]\;\left[\frac{1}{2}\;1\left\{\beta =\frac{1-\omega }{\omega }\right\}+1\left\{0<\beta <\frac{1-\omega }{\omega }\right\}\right], \end{array}$$
  for β>0. In view of (50), (51), (80), (A14), (A19) and (A20), and the monotone convergence theorem,
  Iω(P∥Q)(A21)=Dϕω(P∥Q)(A22)=limα↓0Dfω,α(P∥Q)(A23)=(1−ω)∫0min{1,1−ωω}FP∥Q(logβ)β2dβ−(1−ω)∫1max{1,1−ωω}1−FP∥Q(logβ)β2dβ,
  for ω∈(0,1). We next simplify (A23) as follows:
  - if $\omega \in \left(1,\frac{1-\omega }{\omega }\right)$, then $\frac{1-\omega }{\omega }<1$ and (A23) yields
    (A24) $$\begin{array}{cc} I_{\omega }\left(P∥Q\right) & =\left(1-\omega \right)\int_{0}^{\frac{1-\omega }{\omega }}\;\frac{F_{P∥Q}\left(\log\beta \right)}{\beta^{2}}\;d\beta ; \end{array}$$
  - if $\omega \in \left(0,\frac{1}{2}\right]$, then $\frac{1-\omega }{\omega }\geq 1$ and (A23) yields
    (A25)Iω(P∥Q)=(1−ω)∫01FP∥Q(logβ)β2dβ−(1−ω)∫11−ωω1−FP∥Q(logβ)β2dβ(A26)=(1−ω)∫1∞1−FP∥Q(logβ)β2dβ−(1−ω)∫11−ωω1−FP∥Q(logβ)β2dβ(A27)=(1−ω)∫1−ωω∞1−FP∥Q(logβ)β2dβ,
    where (A26) follows from (95) (or its equivalent from in (73)).
  This completes the proof of (107). Note that, due to (95), the integral representation of $I_{\omega }\left(P∥Q\right)$ in (107) is indeed continuous at $\omega =\frac{1}{2}$.
(6) Triangular discrimination: In view of (36)–(37), the corresponding function ${\tilde{w}}_{f,1}:\left(0,\infty \right)\mapsto R$ in (82) (i.e., with c:=1) can be verified to be given by
  (A28) $$\begin{array}{c} {\tilde{w}}_{f,1}\left(\beta \right)=\frac{4}{{\left(\beta +1\right)}^{2}}\left(1\{\beta \geq 1\}-1\{0<\beta <1\}\right) \end{array}$$
  for β>0. Substituting (80) and (A28) into (83) proves (108) as follows:
  (A29)Δ(P∥Q)=4∫1∞1−FP∥Q(logβ)(β+1)2dβ−∫01FP∥Q(logβ)(β+1)2dβ(A30)=4∫0∞1−FP∥Q(logβ)(β+1)2dβ−∫011(β+1)2dβ(A31)=4∫0∞1−FP∥Q(logβ)(β+1)2dβ−2.
(7) Lin’s measure and the Jensen-Shannon divergence: Let θ∈(0,1) (if θ∈{0,1}, then (39) and (40) imply that $L_{\theta }\left(P∥Q\right)=0$). In view of (41), the application of Theorem 1-(1) with the function $f_{\theta }:\left(0,\infty \right)\mapsto R$ in (42) yields the weight function $w_{f_{\theta }}:\left(0,\infty \right)\mapsto \left[0,\infty \right)$ defined as
  (A32) $$\begin{array}{cc} w_{f_{\theta }}\left(\beta \right) & =\frac{\left(1-\theta )\;\log(\theta \beta +1-\theta \right)}{\beta^{2}}\;\left(1\{\beta \geq 1\}-1\{0<\beta <1\}\right). \end{array}$$
  Consequently, we get
  (A33)Lθ(P∥Q)=(1−θ)∫1∞log(θβ+1−θ)β21−FP∥Q(logβ)dβ−∫01log(θβ+1−θ)β2FP∥Q(logβ)dβ(A34)=(1−θ)∫1∞log(θβ+1−θ)β2dβ−∫0∞log(θβ+1−θ)β2FP∥Q(logβ)dβ(A35)=θlog1θ−(1−θ)∫0∞log(θβ+1−θ)β2FP∥Q(logβ)dβ(A36)=h(θ)−(1−θ)∫0∞1β2logθβ1−θ+1FP∥Q(logβ)dβ
  where (A33) follows from (80), (81) and (A32); for θ∈(0,1), equality (A35) holds since
  (A37) $$\begin{array}{c} \int_{1}^{\infty }\frac{\log(\theta \beta +1-\theta )}{\beta^{2}}\;d\beta =\frac{\theta }{1-\theta }\;\log\frac{1}{\theta }; \end{array}$$
  finally, (A36) follows from (73) where h:[0,1]↦[0,log2] denotes the binary entropy function. This proves (109). In view of (43), the identity in (110) for the Jensen-Shannon divergence follows from (109) with $\theta =\frac{1}{2}$.
(8) Jeffrey’s divergence: In view of (21)–(20), the corresponding weight function $w_{f}:\left(0,\infty \right)\mapsto \left[0,\infty \right)$ in (79) can be verified to be given by
  (A38) $$\begin{array}{c} w_{f}\left(\beta \right)=\left(\frac{\log e}{\beta }+\frac{1}{\beta^{2}}\;\log\frac{\beta }{e}\right)\left(1\{\beta \geq 1\}-1\{0<\beta <1\}\right). \end{array}$$
  Hence, setting c:=loge in (82) implies that
  (A39) $$\begin{array}{c} {\tilde{w}}_{f,c}\left(\beta \right)=\left(\frac{\log e}{\beta }+\frac{\log\beta }{\beta^{2}}\right)\left(1\{\beta \geq 1\}-1\{0<\beta <1\}\right) \end{array}$$
  for β>0. Substituting (80) and (A39) into (83) yields (111).
(9) $E_{\gamma }$ divergence: Let γ≥1, and let $\omega \in \left(0,\frac{1}{2}\right]$ satisfy $\frac{1-\omega }{\omega }=\gamma$; hence, $\omega =\frac{1}{1+\gamma }$. From (53), we get
  (A40) $$\begin{array}{c} E_{\gamma }\left(P∥Q\right)=\left(1+\gamma \right)\;I_{\frac{1}{1+\gamma }}\left(P∥Q\right). \end{array}$$
  The second line in the right side of (107) yields
  (A41) $$\begin{array}{c} I_{\frac{1}{1+\gamma }}\left(P∥Q\right)=\frac{\gamma }{1+\gamma }\int_{\gamma }^{\infty }\frac{1-F_{P∥Q}\left(\log\beta \right)}{\beta^{2}}\;d\beta . \end{array}$$
  Finally, substituting (A41) into the right side of (A40) yields (112).

**Remark** **A1.** *In view of* (95)*, the integral representation for the $\chi^{s}$ divergence in* (104) *specializes to* (100)*,* (105) *and* (106) *by letting s=2 and s=1, respectively.*

**Remark** **A2.** *In view of* (49)*, the first identity for the total variation distance in* (105) *follows readily from* (112) *with γ=1. The second identity in* (106) *follows from* (73) *and* (105)*, and since $\int_{1}^{\infty }\frac{d\beta }{\beta^{2}}=1$.*
